# Supplementary material for: Role of Vitamin D in Preventing and Treating Selected Extraskeletal Diseases—An Umbrella Review
Source: Nutrients. 2020 Mar 31;12(4):969. doi: 10.3390/nu12040969 (PMC7231149; doi:10.3390/nu12040969)
Supplement: Supplementary file 1 [file nutrients-12-00969-s001.pdf]

Role of vitamin D in preventing and treating selected extraskeletal diseases – an umbrella review

Content of supplement

Abbreviations 2

Table S1: Search strategy in PubMed. 3
Table S2: Meta-analyses of RCTs - Asthma 11
Table S3: Meta-analyses of prospective cohort studies – Asthma 15
Table S4: Systematic Reviews of RCTs – Asthma 18
Table S5: Systematic Reviews of prospective cohort studies – Asthma 20
TableS6: Meta-analyses of RCTs – COPD 22
Table S7: Meta-analyses of prospective cohort studies – COPD 22
Table S8: Systematic Reviews of RCTs – COPD.... 23
Table S9: Systematic Reviews of prospective cohort studies – COPD 25
Table S10: Meta-analyses of RCTs – ARI 25
Table S11: Meta-analyses of prospective cohort studies – ARI.... 32
Table S12: Systematic Reviews of RCTs – ARI.... 33
Table S13: Systematic Reviews of prospective cohort studies – ARI 34
Table S14: Meta-analyses of RCTs – Cognitive decline.... 36
Table S15: Meta-analyses of prospective cohort studies – Dementia and cognitive decline 37
Table S16: Systematic Reviews of RCTs – Cognitive decline 41
Table S17: Systematic Reviews of prospective cohort studies – Dementia and cognitive decline.... 43
Table S18: Meta-analyses of RCTs – Depression.... 46
Table S19: Meta-analyses of prospective cohort studies – Depression 49
Table S20: Systematic Reviews of RCTs– Depression 50
Table S21: Systematic Reviews of prospective cohort studies – Depression.... 54
Table S22: Meta-analyses of RCTs – MS.... 56
Table S23: Systematic Reviews of RCTs – MS 61
Table S24: Systematic Reviews of prospective cohort studies – MS.... 65
Table S25: Meta-analysis of prospective cohort studies – T1DM 67
Table S26: Systematic Reviews of RCTs – T1DM.... 68

Figure S1: PRISMA flow diagram – Asthma 4
Figure S2: PRISMA flow diagram – COPD 5
Figure S3: PRISMA flow diagram – ARI.... 6
Figure S4: PRISMA flow diagram – Dementia and cognitive decline 7
Figure S5: PRISMA flow diagram – Depression 8
Figure S6: PRISMA flow diagram – MS 9
Figure S7: PRISMA flow diagram – T1DM.... 10

References 70

Abbreviations

25(OH)D	25-hydroxyvitamin-D
AECOPD	acute exacerbation COPD
aIRR	adjusted incidence rate ratio
ARI	acute respiratory tract infection(s)
ARR	annualised relapse rate
CI	confidence interval
CIS	clinically isolated syndrome
COPD	chronic obstructive pulmonary disease
EDSS	expanded disability status scale
FCP	fasting C-peptide
FeNO	fraction of exhaled nitric oxide
FEV1	forced expiratory volume in 1 second
FFQ	food frequency questionnaire
FIS	fatigue impact scale
HR	hazard ratio
IFN-β	interferon-beta
IL	interleukin
i.m.	intramuscular
IPD	individual patient data
IU	international unit(s)
LRTI	lower respiratory tract infection(s)
MA	meta-analysis
MD	mean difference
MRI	magnetic resonance imaging
MS	multiple sclerosis
MSFC	multiple sclerosis functional composite
NR	not reported
OR	odds ratio
PFT	pulmonary function test
PMS	premenstrual syndrome
RCT	randomised controlled trial
RR	relative risk
RRMS	relapsing-remitting multiple sclerosis
RTI	respiratory tract infection(s)
SCP	stimulated C-peptide
SMD	standardised mean difference
SR	systematic review
T1DM	type 1 diabetes mellitus
TMT	trail making test
URTI	upper respiratory tract infection(s)

Table S1: Search strategy in PubMed[1].

**Study type**	Metaanalysis[tiab] OR „Meta analysis“[tiab] OR „Meta analyses“[tiab] OR Meta-analy*[tiab] OR „systematic review“[tiab] OR systematic[sb][2] OR „Meta-Analysis [mh]"
**Vitamin D**	„vitamin d“ [tiab] OR „vitamin d3“ [tiab] OR „vitamin d2“ [tiab] OR cholecalciferol [tiab] OR ergocalciferol [tiab] OR calcidiol [tiab] OR „25-hydroxyvitamin D“ [tiab] OR 25-hydroxycholecalciferol [tiab] OR hydroxycholecalciferol [tiab] OR calcifediol [tiab] OR calcitriol [tiab] OR „1,25-dihydroxyvitamin D“ [tiab] OR 1,25-dihydroxycholecalciferol [tiab] OR dihydroxycholecalciferol [tiab] OR „1-alpha-hydroxyvitamin D“ [tiab] OR alfacalcidiol [tiab] OR Paricalcitol [tiab] OR „vitamin d“ [mh]
**ARI**	„respiratory tract infection“ [tiab] OR „respiratory tract infections“ [tiab] OR RTI [tiab] OR „respiratory infection“ [tiab] OR „respiratory infections“ [tiab] OR ARI [tiab] OR ARTI [tiab]OR LRTI [tiab] OR URTI [tiab] OR „common cold“ [tiab] OR pneumonia [tiab] OR influenza [tiab] OR flu [tiab] OR „respiratory tract infections“ [mh] OR „respiratory tract diseases“ [mh]
**Asthma**	asthma [tiab] OR „asthma“ [mh]
**COPD**	COPD [tiab] OR „chronic obstructive pulmonary disease“ [tiab] OR „chronic obstructive lung disease“ [tiab] OR exacerbation [tiab] OR exacerbations [tiab] OR emphysema [tiab] OR bronchitis [tiab] OR „pulmonary disease, chronic obstructive“ [mh]
**Dementia and cognitive decline**	dementia [tiab] OR dementias [tiab] OR alzheimer [tiab] OR alzheimers [tiab] OR alzheimer´s [tiab] OR cognitive [tiab] OR cognition [tiab] OR „lewy body disease" [tiab] OR „frontotemporal lobar degeneration" [tiab] OR neurodegenerative [tiab] OR neurodegeneration [tiab] OR dementia [mh] OR „cognitive dysfunction" [mh]
**Depression**	depression [tiab] OR depressions [tiab] OR depressive [tiab] OR „affective disorder“ [tiab] OR „affective disorders“ [tiab] OR mood [tiab] OR „depression" [mh] OR „depressive disorder" [mh]
**T1DM**	„type 1 diabetes“ [tiab] OR „diabetes mellitus type 1“ [tiab] OR „Diabetes Mellitus, Type 1" [mh]
**MS**	„multiple sclerosis“ [tiab] OR „neuroinflammatory autoimmune disease“ [tiab] OR „multiple sclerosis“ [mh]

[1] Identical search terms for the systematic literature searches across the Cochrane Reviews library
[2] PubMed systematic reviews filter before December 2018

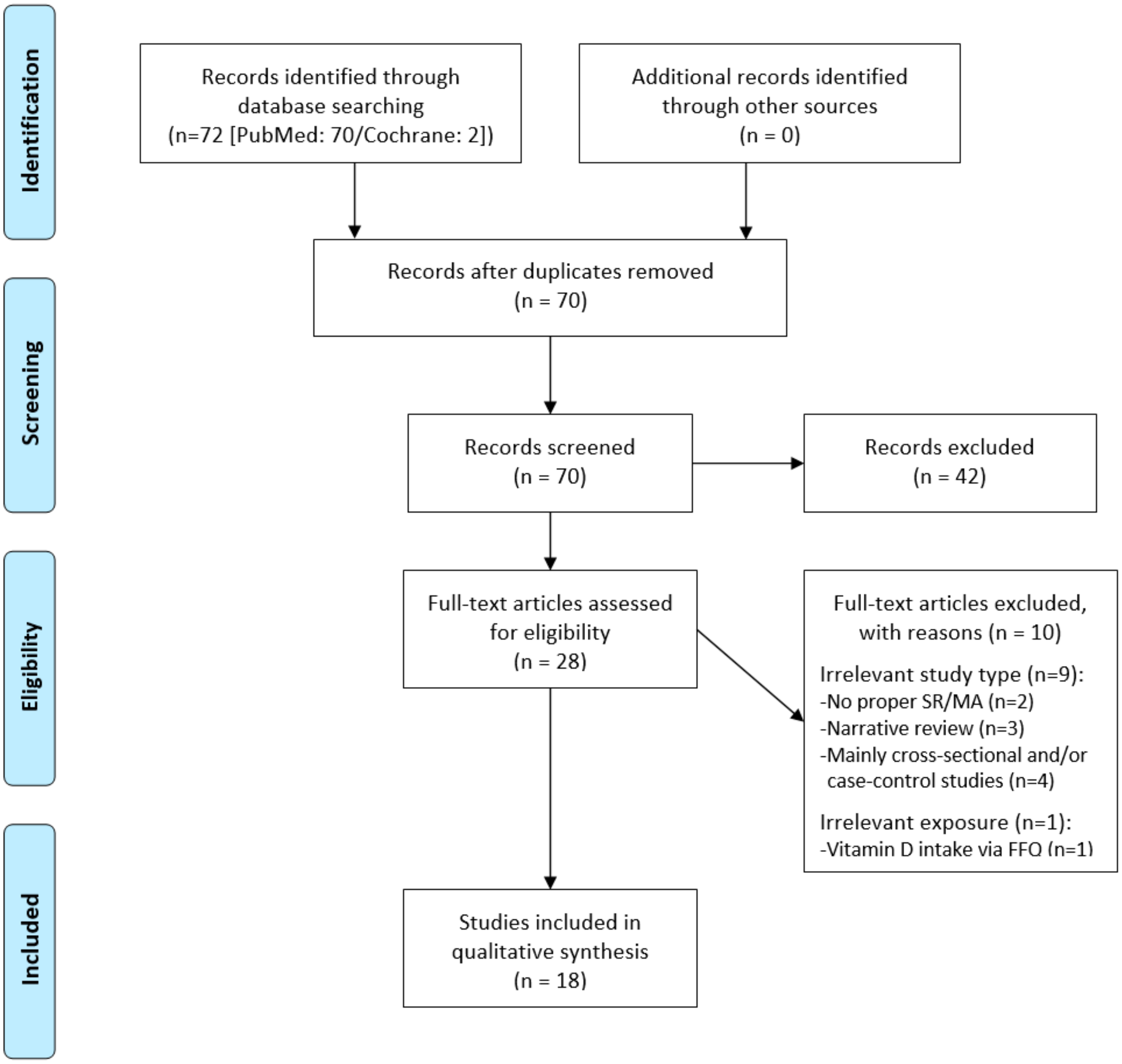

Figure S1: PRISMA flow diagram – Asthma

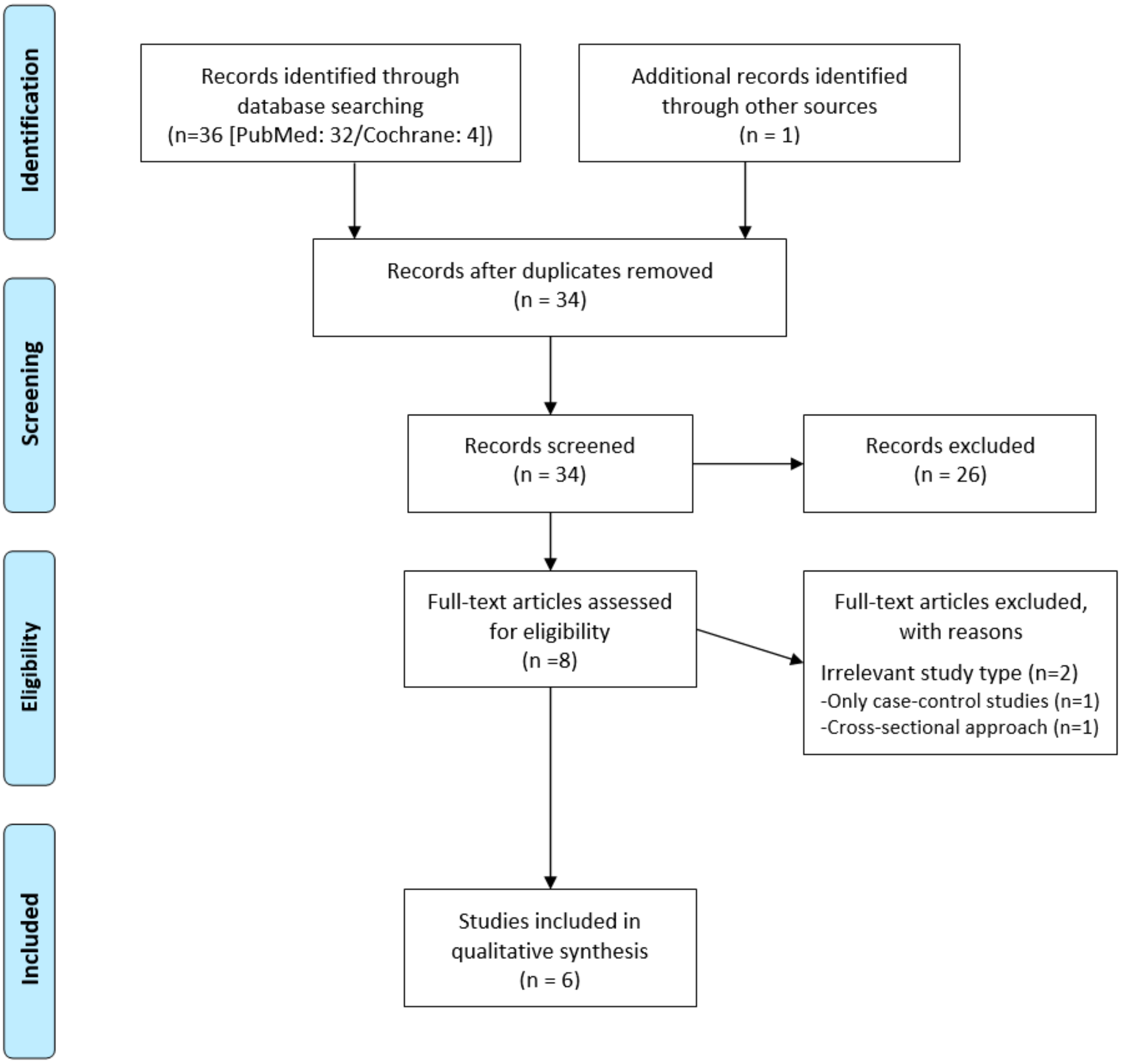

Figure S2: PRISMA flow diagram – COPD

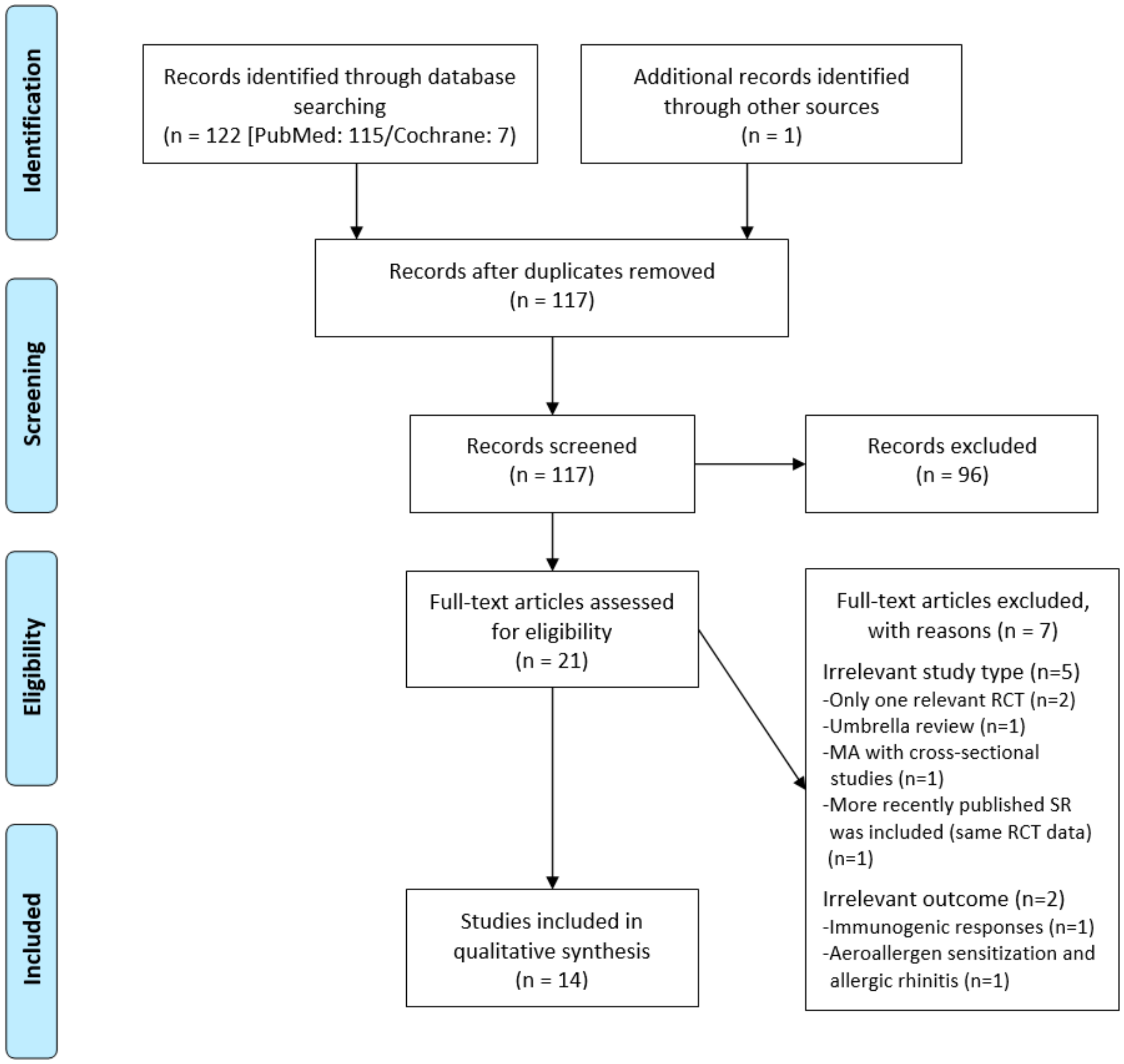

Figure S3: PRISMA flow diagram – ARI

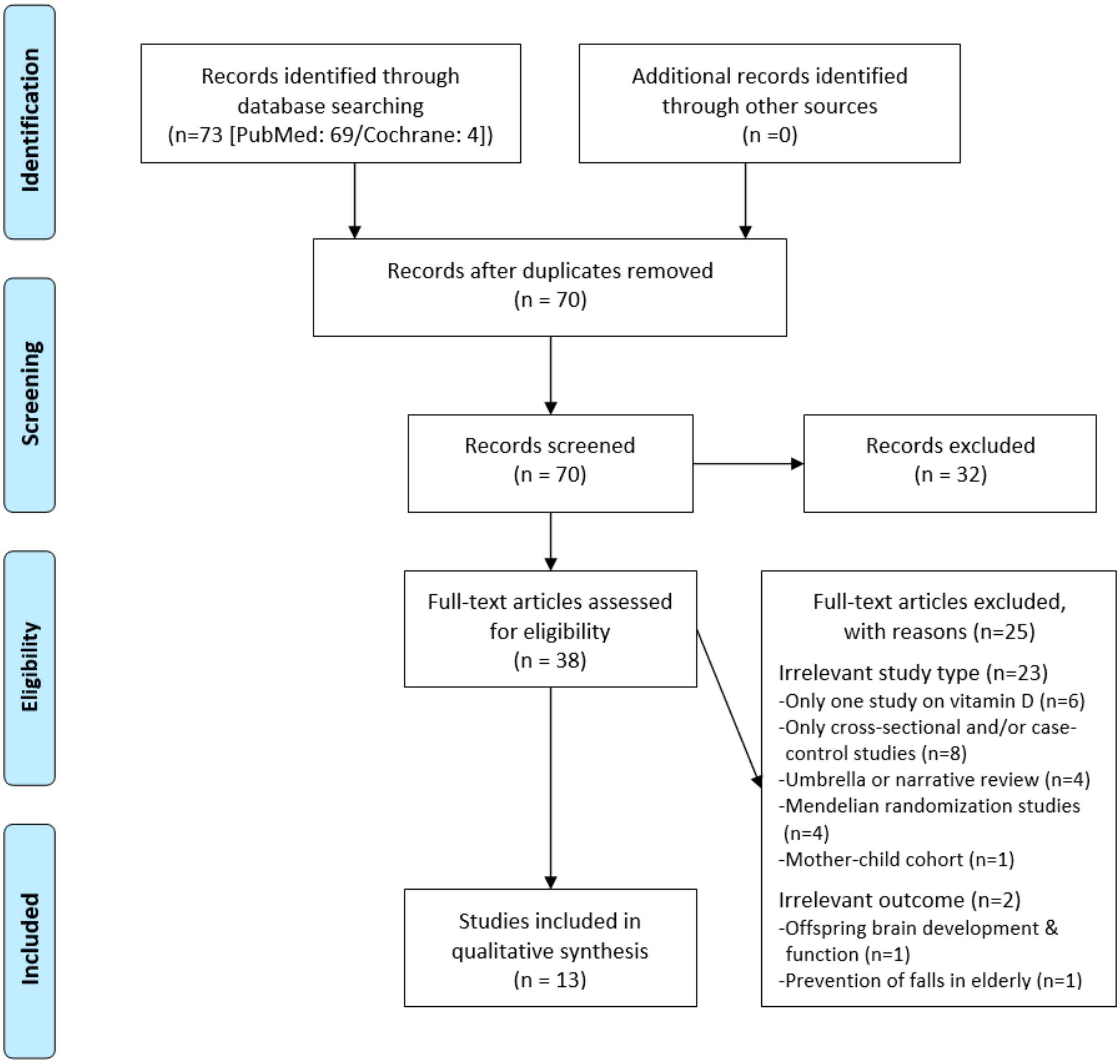

Figure S4: PRISMA flow diagram – Dementia and cognitive decline

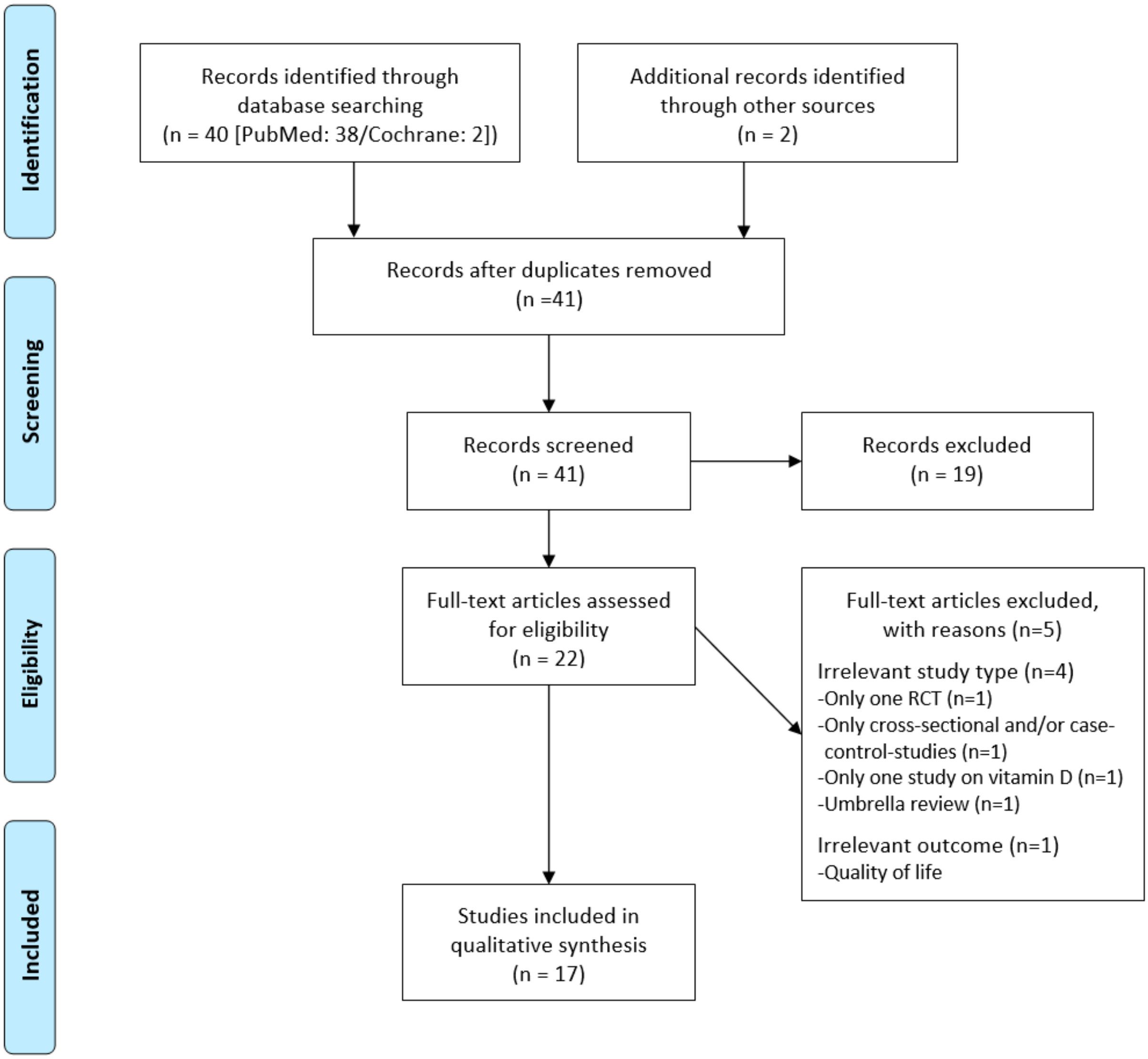

Figure S5: PRISMA flow diagram – Depression

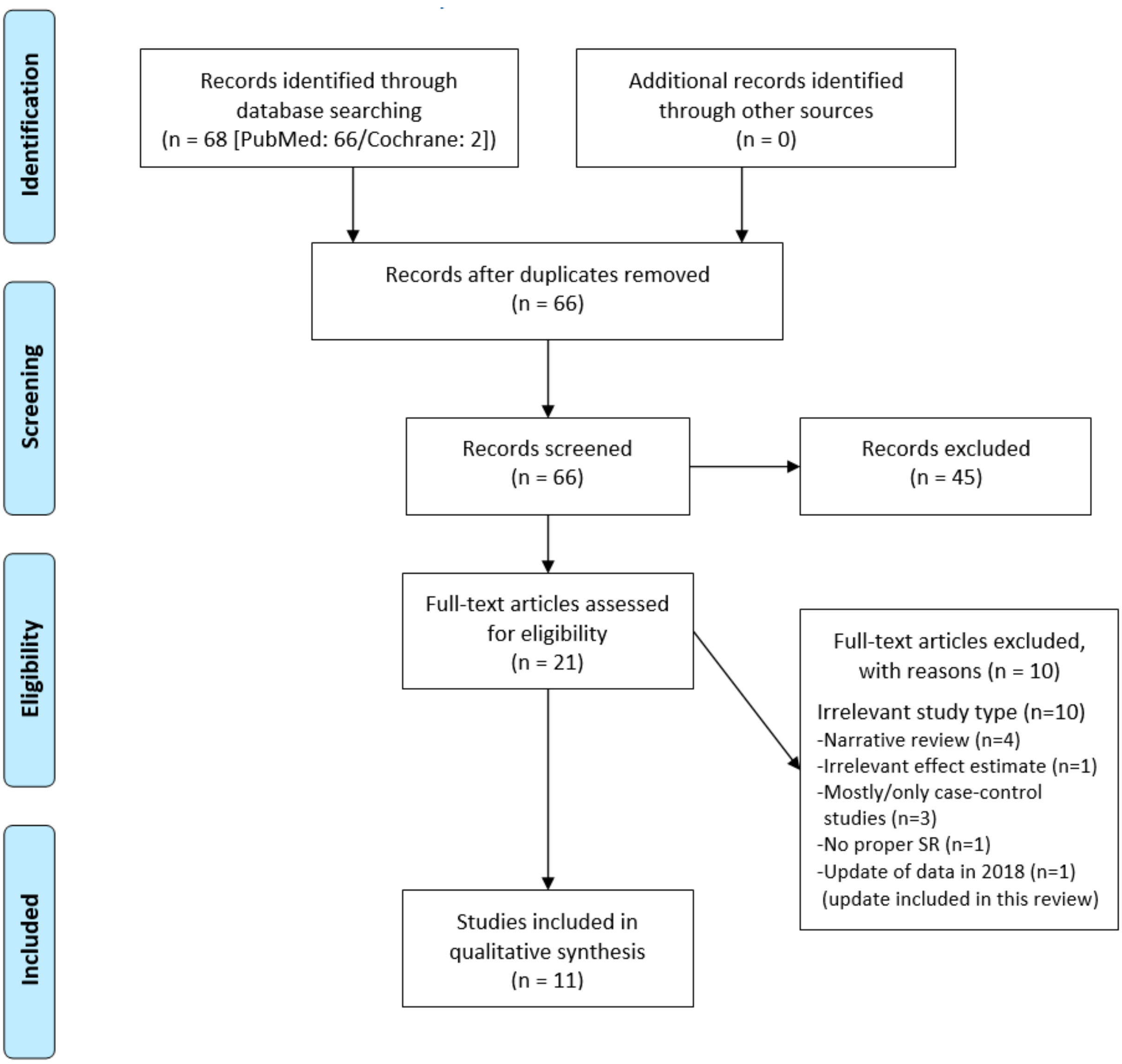

Figure S6: PRISMA flow diagram – MS

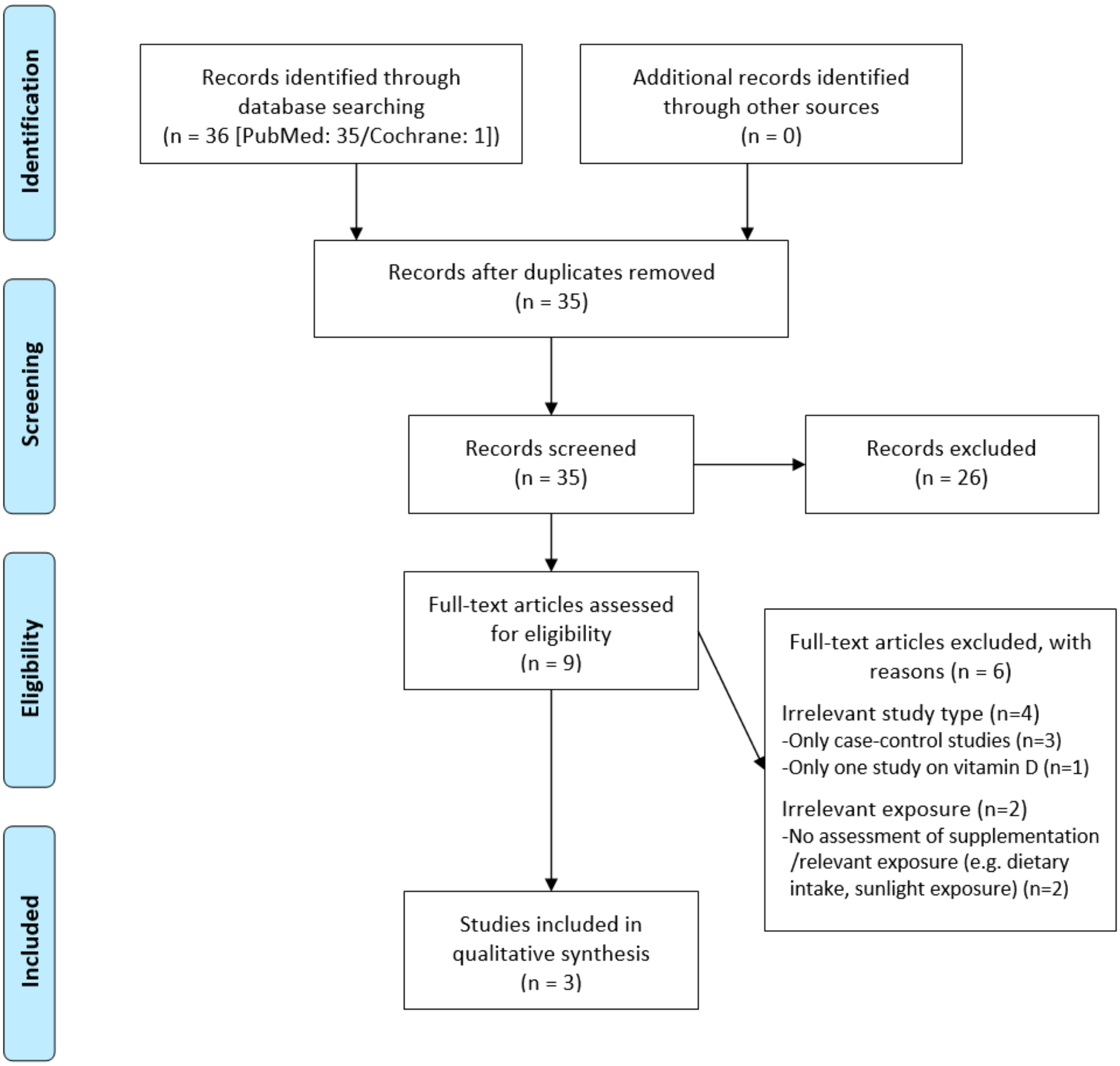

Figure S7: PRISMA flow diagram – T1DM

Table S2: Meta-analyses of RCTs - Asthma

**Meta-analyses of RCTs**							
**Author, year**	**Included studies (n)**	**Participants (n), gender, age**	**Vitamin D dose**	**Control/ Comparator**	**Outcome**	**Results/ Summary statistics (95% CI)**	**AMSTAR 2**
Jolliffe et al. 2017 [1]	7	n= 955 participants with asthma (297 children, 658 adults)<br><br>Both sexes<br>Age: 1.6-85 yr	Vitamin D_3<br>1200 IU/d (4 mth)<br>500 IU/d (6 mth)<br>100,000 IU bolus, then 4000 IU/d (28 wk)<br>120,000 IU bolus once/2 mth (1 yr)<br>800 IU/d, first 2 mth (6 mth)<br>2000 IU/d (15 wk)<br>100,000 IU bolus, then 400 IU/d (6 mth)	Placebo<br>Placebo<br>Placebo<br>Placebo<br>Placebo<br>Placebo<br>400 IU/d	Rate of asthma exacerbations requiring treatment with systemic corticosteroids	Overall results:<br>aIRR 0.74 (0.56, 0.97)	No assessment, because IPD-MA
					Proportion of people with ≥ 1 exacerbation treated with systemic corticosteroids (secondary outcome)	Adjusted OR 0.75 (0.51, 1.09)	
					Asthma exacerbation resulting in emergency department attendance or hospital admission or both (secondary outcome)	Adjusted OR 0.46 (0.24, 0.91)	
					Asthma exacerbation as defined in primary trial (secondary outcome)	Adjusted OR 0.81 (0.58, 1,11)	
Vahdaninia et al. 2017 [2]	3	n= 1493 mother-child-pairs/ 337 events<br><br>Both sexes<br>Age: NR	Vitamin D_3<br>2400 IU/d (3.5-4 mth + 1 wk)<br>200,000 IU bolus (vitamin D_3) or 800 IU/d (until	Placebo<br>No treatment	Asthma or wheeze incidence assessed at 3 years of age	RR 0.81 (0.67, 0.98)	Moderate

Meta-analyses of RCTs							
**Author, year**	**Included studies (n)**	**Participants (n), gender, age**	**Vitamin D dose**	**Control/ Comparator**	**Outcome**	**Results/ Summary statistics (95% CI)**	**AMSTAR 2**
			delivery vitamin D_2;<br>3 mth + 1 wk)<br><br>Vitamin D<br>4000 IU/d (5-7.5 mth)	 <br><br>No treatment			
Martineau et al. 2016 [3]	9	n= 1093 participants with asthma (435 children, 658 adults)<br><br>Majority of participants: mild/moderate asthma<br><br>25(OH)D concentrations at baseline: 48-89 nmol/l; small minority: < 25 nmol/l<br><br>Both sexes<br>Age: 1 - ≥18 yr	Vitamin D_3<br>100,000 IU bolus, then 4000 IU/d (28 wk)<br><br>100,000 IU bolus, then 400 IU/d (6 mth)<br><br>1000 IU/d (12 mth)<br><br>1000 IU/wk (3 mth)<br><br>500 IU/d (6 mth)<br><br>120,000 IU/2 mth (12 mth)<br><br>800 IU/d (2 mth)<br><br>1200 IU/d (24 wk)<br><br>60,000 IU/mth (6 mth)	Placebo<br><br>Placebo, then 400 IU/d<br><br>Placebo<br><br>Placebo<br><br>Placebo<br><br>Placebo<br><br>Placebo<br><br>Placebo<br><br>Placebo	Rate ratio of exacerbations requiring treatment with systemic corticosteroids (primary outcome)	RR 0.64 (0.46, 0.90)<br>(3 studies)	High
					≥ 1 exacerbations requiring visits to an emergency department or hospitalisation (secondary outcome)	OR 0.39 (0.19, 0.78)<br>(7 studies)	
					People with ≥ 1 exacerbation (secondary outcome)	OR 0.53 (0.28, 0.99)<br>(7 studies)	
					Asthma control test (secondary outcome)	MD -0.08 (-0.70, 0.54)<br>(3 studies)	
					FEV1 (% of predicted value) (secondary outcome)	MD 0.48 (0.93, 1.89)<br>(4 studies)	
Luo et al. 2015 [4]	7	n= 903 participants with asthma	Vitamin D<br>(frequency: NR)		Rate of asthma exacerbation	RR 0.66 (0.32, 1.37)<br>(3 studies)	Moderate

Meta-analyses of RCTs							
**Author, year**	**Included studies (n)**	**Participants (n), gender, age**	**Vitamin D dose**	**Control/ Comparator**	**Outcome**	**Results/ Summary statistics (95% CI)**	**AMSTAR 2**
		3 studies in children (mean age 9 yr), 4 in adults (mean age 40-55 yr)<br><br>25(OH)D concentrations at baseline: 49.8-60 nmol/l<br><br>Both sexes<br>Age: 9-59 yr	1000 IU +calcium (6mth)<br><br>1000 IU subcutaneous,12 mth)<br><br>650 IU (subcutaneous, 12 mth)<br><br>100,000 IU, then 4000 IU (oral, 28 wk)<br><br>60,000 IU (oral, 6 mth)<br><br>40,000 IU (oral, 9 wk)<br><br>120,000 IU (oral, 12 mth)	Calcium + Placebo<br><br>Placebo<br><br>Placebo<br><br>Placebo<br><br>Placebo<br><br>Placebo<br><br>Placebo	FEV1 (% of predicted value)	SMD -0.02 (-0.15, 0.11) (4 studies)	
					FeNO	SMD -0.02 (-0.16, 0.12) (2 studies)	
					Asthma control test	SMD -0.05 (-0.17, 0.06) (2 studies)	
Riverin et al. 2015 [5]	8	n= 573 children diagnosed with asthma<br><br>Both sexes<br>Age: 3-18 yr	<u>Vitamin D_3</u><br>650 IU/d (12 mth)<br><br>60,000 IU/mth (6 mth)<br><br>500 IU/d (6 mth)<br><br>1000 IU/d (12 mth)<br><br>500 IU/d (6 mth)<br><br>1200 IU/d (4 mth)	Placebo<br><br>No treatment<br><br>Placebo<br><br>No treatment<br><br>Placebo<br><br>No treatment	Emergency department visits and/or hospitalisation admissions for asthma exacerbations	Significantly less emergency department visits for children treated with vitamin D (1 study, n=100)	High
					Rate of asthma exacerbations (secondary outcome)	RR 0.41 (0.27, 0.63) (3 studies)	
					Asthma symptom scores (secondary outcome)	SMD 0.10 (-0.59, 0.80) (3 studies)	

Meta-analyses of RCTs							
**Author, year**	**Included studies (n)**	**Participants (n), gender, age**	**Vitamin D dose**	**Control/ Comparator**	**Outcome**	**Results/ Summary statistics (95% CI)**	**AMSTAR 2**
			1000 IU/wk (12 mth)<br><br>600 IU/d (1 mth)	Placebo<br><br>Placebo	FEV1 (% of predicted value) (secondary outcome)	MD 0.00 (-3.17, 3.1)	
Xiao et al. 2015 [6]	2	n= 478 children with newly diagnosed asthma<br><br>Both sexes<br>Age: 10-12 yr	Vitamin D_3<br>1200 IU/d (4 mth)<br><br>500 IU/d (6 mth)	NR	Asthma exacerbation triggered by respiratory infections	RR 0.28 (0.12, 0.64)	High
Fares et al. 2015 [7]	2	n=102 children with asthma<br><br>Both sexes<br>Age: 5-18 yr	Vitamin D_3<br>1000 IU/week (1 yr)<br><br>500 IU (frequency: NR; 6 mth)	Placebo	FEV1 (% of predicted value)	MD -0.54 (-5.28, 4.19) (2 studies)	High
Pojsupap et al. 2015 [8]	3	n= 578 children and adolescents<br><br>2/3 studies included asthmatic patients. One study enrolled 430 school children; 26%	Vitamin D_3<br>1200 IU/d (15-17 wk)<br><br>500 IU/d (26 wk)<br><br>60,000 IU/mth (26 wk)	Placebo	Asthma exacerbations	RR 0.41 (0.27, 0.63) (3 studies)	Moderate

Meta-analyses of RCTs							
**Author, year**	**Included studies (n)**	**Participants (n), gender, age**	**Vitamin D dose**	**Control/ Comparator**	**Outcome**	**Results/ Summary statistics (95% CI)**	**AMSTAR 2**
		diagnosed with asthma<br><br>Both sexes<br>Age: 5-18 years					

Table S3: Meta-analyses of prospective cohort studies – Asthma

Meta-analyses of prospective cohort studies							
**Author, year**	**Included studies (n)**	**Participants (n), gender, age**	**Vitamin D dose**	**Control/ Comparator**	**Outcome**	**Results/ Summary statistics (95% CI)**	**AMSTAR 2**
Shen et al. 2018 [9]	8	n= 35,000 mother-child-pairs<br><br>asthma incidence in adulthood (1 study)<br><br>Both sexes<br>Age: NR	25(OH)D in maternal blood or cord blood	-	Asthma incidence assessed at > 5 years of age	Highest vs. lowest category of 25(OH)D (8 studies): OR 0.96 (0.79, 1.18)	High
						≥ 75 nmol/l vs. < 50 nmol/l (5 studies): OR 1.11 (0.92, 1.33)	
					Asthma incidence assessed at ≤ 5 years of age	Highest vs. lowest category of 25(OH)D (6 studies): OR 0.81 (0.65, 1.01)	
						≥ 75 nmol/l vs. < 50 nmol/l (6 studies): OR 0.93 (0.85, 1.03)	

Meta-analyses of prospective cohort studies							
**Author, year**	**Included studies (n)**	**Participants (n), gender, age**	**Vitamin D dose**	**Control/ Comparator**	**Outcome**	**Results/ Summary statistics (95% CI)**	**AMSTAR 2**
Pacheco-González et al. 2018 [10]	14	n= 33,521 mother-child-pairs<br><br>Both sexes<br>Age: NR	25(OH)D in maternal blood or cord blood	-	Asthma incidence in childhood assessed between 3-14 years of age	Highest vs. lowest category of 25(OH)D:<br>OR 0.91 (0.78, 1.06)	High
Song et al. 2017 [11]	15<br>(14 birth cohorts and 1 nested case-control study)<br><br>Meta-analysis of 12 cohort studies	n= 12,758 mother-child-pairs/ 1795 events<br><br>Both sexes<br>Age: ≤ 18 yr	25(OH)D in maternal blood or cord blood<br><br>mean maternal 25(OH)D ranged from 44 to 74 nmol/l	-	Incidence of childhood asthma	Highest vs. lowest category of 25(OH)D (12 studies):<br>RR 0.87 (0.75, 1.02)<br>Per 10 nmol/l increase of maternal 25(OH)D levels (7 studies):<br>RR 0.99 (0.95, 1.02)<br><br>An U-shaped dose-response relationship was found between 25(OH)D levels and risk of childhood asthma, with the lowest risk at approx. 70 nmol/l of 25(OH)D, and remained protective until a concentration of about 130 nmol/l. Further increase tended to be a risk factor for childhood asthma.	High
Feng et al. 2017 [12]	10	n= 8871 mother-child-	25(OH)D in maternal blood or cord blood	-	Asthma incidence in childhood assessed at 4-14 years of age	Highest vs. lowest category of 25(OH)D (8 studies):<br>OR 0.84 (0.70, 1.01)	High

Meta-analyses of prospective cohort studies							
**Author, year**	**Included studies (n)**	**Participants (n), gender, age**	**Vitamin D dose**	**Control/ Comparator**	**Outcome**	**Results/ Summary statistics (95% CI)**	**AMSTAR 2**
		pairs/ 1494 events<br><br>Both sexes<br>Age: NR				Each 10 nmol/l increment in 25(OH)D (8 studies):<br>OR 0.99 (0.97, 1.02)	
Wei et al. 2016 [13]	4	n= 3666 mother-child-pairs<br>Both sexes<br>Age: NR	25(OH)D in maternal blood or cord blood (3 studies) or intake of vitamin D via food or supplement (1 study)	-	Asthma incidence in childhood assessed at 5-6 years of age	Highest vs. lowest category of 25(OH)D:<br>OR 0.98 (0.94, 1.02)	High
Man et al. 2015 [14]	4	n=1291 events<br><br>Both sexes<br>Age: 0-<12 yr	25(OH)D (maternal blood or cord blood)<br><br>Deficiency: <50 nmol/l<br><br>Insufficiency: < 75 nmol/l	-	Incidence of childhood asthma	Vitamin D deficiency (4 studies):<br>RR 1.57 (1.26, 2.02)<br><br>Vitamin D insufficiency (2 studies):<br>RR 1.25 (1.01, 1.55)	Low
Cassim et al. 2015 [15]	11	n= range: 14 to 6487 mother-child-pairs<br><br>Both sexes<br>Age: 1-20 yr	25(OH)D in maternal blood or cord blood<br><br>25(OH)D levels in childhood (4 cohorts)	Asthma incidence	Parental reports, physician diagnosis of asthma, use of inhaler medication for asthma	No association between 25(OH)D levels and asthma incidence (studies= 5/6). Increasing maternal serum 25(OH)D during pregnancy increased the risk of asthma in offspring at age of 9 (1 study).<br><br>Investigated incident asthma in children with serum 25(OH)D measured in childhood found no association. (3/4 studies)	Very low

Meta-analyses of prospective cohort studies							
**Author, year**	**Included studies (n)**	**Participants (n), gender, age**	**Vitamin D dose**	**Control/ Comparator**	**Outcome**	**Results/ Summary statistics (95% CI)**	**AMSTAR 2**
			25(OH)D levels in childhood	Asthma exacerbations requiring hospitalisation and treatment with oral steroids		High vs. low 25(OH)D levels (2 cohort studies and 2 cross-sectional studies): RR 0.64 (0.50, 0.81)	

Table S4: Systematic Reviews of RCTs – Asthma

Systematic Reviews of RCTs							
**Author, year**	**Included studies (n)**	**Participants (n), gender, age**	**Vitamin D dose**	**Control/ Comparator**	**Outcome**	**Results**	**AMSTAR 2**
Shen et al. 2018 [9]	2	n= 1499 mother-child-pairs<br><br>Both sexes<br>Age: NR	Vitamin D_3<br>2400 IU/d<br><br>4000 IU/d + multivitamin with 400 IU	Placebo<br><br>Placebo + multivitamin with 400 IU vitamin D	Asthma incidence assessed from birth to 3 years of age	Non-significant trends of vitamin D supplementation during pregnancy on preventing the development of offspring asthma.	High
Fares et al. 2015 [7]	4	n= 149 children with asthma<br><br>Both sexes<br>Age:5-18 yr	Vitamin D_3<br>1000 IU/wk (1yr)<br><br>1000 IU/d (1yr)<br><br>600 IU/d (+multivitamin supplement) (4wk)	No treatment<br><br>Placebo<br><br>Placebo	Asthma symptoms	Improvement in asthma symptoms in the vitamin D supplemented study group, but no statistically significant difference between the groups (3 studies).	High

Systematic Reviews of RCTs							
**Author, year**	**Included studies (n)**	**Participants (n), gender, age**	**Vitamin D dose**	**Control/ Comparator**	**Outcome**	**Results**	**AMSTAR 2**
			500 IU (frequency: NR; 6 mth)	Placebo		No effect of vitamin D supplementation on the asthma symptom score (1 study).<br><br>The RCTs used different instruments to measure the outcome, therefore results were not pooled in a meta-analysis.	
Pojsupap et al. 2015 [8]	5	n= 625 children and adolescents 4/5 studies included asthmatic patients. One study enrolled 430 school children; 26% diagnosed with asthma<br><br>Both sexes Age: 5-18 years	Vitamin D_3<br>600 IU/d (4 wk)<br><br>1200 IU/d (15-17 wk)<br><br>500 IU/d (26 wk)<br><br>1000 IU/d (26, 52 wk)<br><br>60,000 IU/month (26 wk)	Placebo	PFT	Greater improvements in PFTs for the vitamin D group (2/4 studies)	Moderate
					Asthma symptom scores	Report of pre- and postintervention (2/3 studies). No difference in symptom score between groups (1/2 studies) and a greater reduction in asthma symptoms in the placebo group (1/2 studies).	

Table S5: Systematic Reviews of prospective cohort studies – Asthma

**Systematic Reviews of prospective cohort studies**							
**Author, year**	**Included studies (n)**	**Participants (n), gender, age**	**Vitamin D dose**	**Control/ Comparator**	**Outcome**	**Results**	**AMSTAR 2**
Jat and Khairwa 2017 [16]	3 (birth cohorts)	n= 3991 mother-child-pairs	25(OH)D in maternal blood or cord blood	-	asthma incidence assessed at age of 4 to 14	Inverse association between 25(OH)D concentrations and asthma/severe asthma at age of 4; no association between 25(OH)D and severe asthma at age of 8 (1 study).<br><br>No association between cord blood vitamin D levels and incidence of asthma at age of 5 for insufficiency and deficiency compared to sufficiency (1 study)<br><br>In a pregnancy cohort asthma at age of 14 was not related to vitamin D levels.	Moderate
Cassim et al. 2015 [15]	4 (2 cohort studies, 2 cross-sectional studies)	n= range: 226 to 1024 mother-child-pairs<br><br>Both sexes Age: 1-20 yr	25(OH)D levels in childhood	-	Asthma exacerbations requiring hospitalisation and treatment with oral steroids	High vs. low 25(OH)D levels: RR 0.64 (0.50, 0.81)	Very low

Systematic Reviews of prospective cohort studies							
**Author, year**	**Included studies (n)**	**Participants (n), gender, age**	**Vitamin D dose**	**Control/ Comparator**	**Outcome**	**Results**	**AMSTAR 2**
Harvey et al. 2014 [17]	3	n= 2234 mother-child-pairs<br><br>Both sexes<br>Age: NR	25(OH)D in maternal blood or cord blood	-	Asthma incidence	Cord blood levels of 25(OH)D had no association with incident asthma at age of 5.<br><br>No association between maternal 25(OH)D and offspring asthma at age of 4-6.<br><br>Children whose mothers had a 25(OH)D level in pregnancy of > 75 nmol/l had an increased risk of asthma at age of 9 compared to children whose mothers had a level < 30 nmol/l.	High
Rajabbik et al. 2014 [18]	3	n= 4684 children<br><br>Both sexes<br>Age: 8-15.5 yr	Serum 25(OH)D	-	Asthma incidence	Low serum 25(OH)D level was associated with an increased risk of developing asthma late in childhood (2 studies), while one found no association.	High

TableS6: Meta-analyses of RCTs – COPD

**Meta-analyses of RCTs**							
**Author, year**	**Included studies (n)**	**Participants (n), gender, age**	**Vitamin D dose**	**Control/ Comparator**	**Outcome**	**Results/ Summary statistics (95% CI)**	**AMSTAR 2**
Jolliffe et al. 2019 [19]	3 (individual participant data)	n=472<br><br>Both sexes (66.7% men)<br><br>Age: 40-86 yr	Vitamin D_3<br>100,000 IU/mth (12 mth)<br><br>120,000 IU/2 mth (12 mth)<br><br>1200 IU/d (6 mth)	NR	Rate of COPD exacerbations	Overall results:<br>aIRR 0.94 (0.78, 1.13)	Low
						Baseline 25(OH)D levels < 25 nmol/l (87 participants):<br>aIRR 0.55 (0.36, 0.84)	
						Baseline 25(OH)D levels ≥ 25 nmol/l (382 participants):<br>aIRR 1.04 (0.85, 1.27)	

Table S7: Meta-analyses of prospective cohort studies – COPD

**Meta-analyses of prospective cohort studies**							
**Author, year**	**Included studies (n)**	**Participants (n), gender, age**	**Vitamin D dose**	**Control/ Comparator**	**Outcome**	**Results/ Summary statistics (95% CI)**	**AMSTAR 2**
Zhu et al. 2016 [20]	2 cohort and 5 case-control studies	n= 2091 COPD patients<br><br>Both sexes<br>Age: NR	25(OH)D	-	Severity of COPD	25(OH)D level of severe-very severe COPD patients vs. mild-moderate COPD patients:<br>SMD -0.87 (-1.51, -0.22).	High
	2 cohort and 3 case-control studies	n= 278 AECOPD patients & 563 stable COPD patients<br><br>Both sexes<br>Age: NR			COPD exacerbations	25(OH)D level of AECOPD patients vs. stable COPD patients:<br>SMD -0.43 (-0.70, -0.15)	

Meta-analyses of prospective cohort studies							
**Author, year**	**Included studies (n)**	**Participants (n), gender, age**	**Vitamin D dose**	**Control/ Comparator**	**Outcome**	**Results/ Summary statistics (95% CI)**	**AMSTAR 2**
Zhu et al. 2015 [21]	8	n=6313 COPD patients/2418 controls* (for 3 studies data NR)<br><br>Both Sexes<br>Age: NR	25(OH)D<br><br>Deficiency: 25(OH)D <50 nmol/l	-	COPD	25(OH)D levels of COPD patients vs. controls (4 studies): SMD 0.19 (-0.13, 0.51)	Moderate
						Deficiency rates COPD patients vs. controls (4 studies): RR 0.96 (0.75, 1.21)	
					Severity of COPD	Deficiency rates of 25(OH)D (mild COPD vs. moderate/ severe COPD) (3 studies): RR 0.72 (0.63, 0.83)	
						Deficiency rates of 25(OH)D (moderate COPD vs. severe COPD) (n=4): RR 0.74 (CI 0.56, 0.98)	

Table S8: Systematic Reviews of RCTs – COPD

Systematic Reviews of RCTs							
**Author, year**	**Included studies (n)**	**Participants (n), gender, age**	**Vitamin D dose**	**Control/ Comparator**	**Outcome**	**Results**	**AMSTAR 2**
Ferrari et al. 2018 [22]		510 COPD patients<br><br>Both Sexes<br>Age: NR	Vitamin D_3<br>Dose: NR	NR	Exacerbation frequency	Vitamin D_3 supplementation reduced the risk of moderate and severe exacerbation in COPD patients with 25(OH)D levels <50 nmol/l or < 25nmol/l (2/3 studies).	Very low

Systematic Reviews of RCTs							
**Author, year**	**Included studies (n)**	**Participants (n), gender, age**	**Vitamin D dose**	**Control/ Comparator**	**Outcome**	**Results**	**AMSTAR 2**
Autier et al. 2017 [23]	3	n=512 COPD patients<br><br>Both Sexes<br>Age: NR	Vitamin D<br>very high doses<br>(7 d – 1 yr)	Calcitriol<br>10 IU/d or placebo	Respiratory function and time to first exacerbation	No effect of vitamin D supplementation on the investigated outcome parameters.	Low
Zhu et al. 2015 [21]	5	n=596 COPD patients (300 vitamin D, 296 with placebo)<br><br>Both sexes<br>Age: NR	Vitamin D<br>100,000 IU/mth (6 mth)<br><br>120,000 IU/2 mth (6 mth)<br><br>2000 IU/d (6 wk)<br><br>100,000 IU/mth (1 yr)<br><br>100,000 IU/mth (1 yr)	Placebo	Exacerbations, maximal oxygen uptake, inspiratory Muscle strength	Beneficial effect of vitamin D intake in COPD patients (4/5 studies).<br><br>Inhibition of exacerbations and improvement of FEV1 within severe COPD patients or patients with baseline 25(OH)D levels <50 nmol/l.<br><br>Improvements in inspiratory muscle strength and maximal oxygen uptake.	Moderate
Autier et al. 2014 [24]	1	n= 182<br><br>Sex and age: NR	Vitamin D<br>3560 IU/d (12 mth)	Placebo	Exacerbation	No improvement.	Low

Table S9: Systematic Reviews of prospective cohort studies – COPD

Systematic Reviews of prospective cohort studies							
**Author, year**	**Included studies (n)**	**Participants (n), gender, age**	**Vitamin D dose**	**Control/ Comparator**	**Outcome**	**Results**	**AMSTAR 2**
Ferrari et al. 2018 [22]	6	n= 2473 COPD patients<br><br>Both sexes<br>Age: NR	25(OH)D	-	Exacerbation frequency	No association between exacerbation frequency and vitamin D levels (majority of studies).	Very low
Autier et al. 2014 [24]	2	n=1070<br><br>Both sexes<br>Age: NR	25(OH)D	-	Exacerbation	Data from two studies of patients with COPD showed decreases in risk of exacerbation with high 25(OH)D concentrations	Low

Table S10: Meta-analyses of RCTs – ARI

Meta-analyses of RCTs							
**Author, year**	**Included studies (n)**	**Participants (n), gender, age**	**Vitamin D dose**	**Control/ Comparator**	**Outcome**	**Results/ Summary statistics (95% CI)**	**AMSTAR 2**
Martineau et al. 2019 [25]	25	n= 10,933 participants<br><br>Both sexes<br>Age: 0-95 yr	Vitamin D_3<br>2000 IU/d (3 mth)<br><br>1200 IU/d (4 mth)<br><br>100,000 IU bolus (3 mth)<br><br>400 IU/d (6 mth)	 Placebo<br><br>Placebo<br><br>Placebo<br><br>Placebo	Proportion of participants experiencing at least one ARI	Adjusted OR 0.88 (0.81, 0.96) (25 studies)	No assessment, because IPD-MA
					ARI rate	Adjusted incidence rate ratio 0.96 (0.92, 0.997) (25 studies)	
					Time to first ARI	Adjusted HR 0.95 (0.89, 1.01) (8 studies)	

Meta-analyses of RCTs							
**Author, year**	**Included studies (n)**	**Participants (n), gender, age**	**Vitamin D dose**	**Control/ Comparator**	**Outcome**	**Results/ Summary statistics (95% CI)**	**AMSTAR 2**
			500 IU/d (6 mth)	Placebo			
			1400 IU/wk (6 mth)	Placebo			
			100,000 IU bolus/mth (1y)	Placebo			
			100,000 IU bolus/3 mth (1.5 yr)	Placebo			
			300 IU/d (7 wk)	Placebo			
			2x 200,000 IU/mth, then 100,000 IU/mth (1,5 yr)	Placebo			
			4000 IU/d (1yr)	Placebo			
			1000 IU/d (6 mth)	Placebo			
			1000 IU/d (average: 13 mth)	Placebo			
			60,000 IU/mth or 30,000 IU/mth (1 yr)	Placebo			
			10,000 IU/wk (8 wk)	Placebo			
			2000 IU/d (2 mth)	Placebo			
			Mothers: 1000 or 2000 IU/d Infants: 400 or 800 IU/d (9 mth: 3 mth	Placebo			

Meta-analyses of RCTs							
**Author, year**	**Included studies (n)**	**Participants (n), gender, age**	**Vitamin D dose**	**Control/ Comparator**	**Outcome**	**Results/ Summary statistics (95% CI)**	**AMSTAR 2**
			in pregnancy, 6 mth in infants)				
			120,000 IU/2 mth (1yr)	Placebo			
			Older adults:<br>96,000 IU/2 mth + 400 IU/d (1 yr)<br>Carers:<br>120,000 IU/2 mth (1 yr)	Placebo+ 400 IU/d<br><br>Carers:<br>Placebo			
			20,000 IU/wk (17 wk)	Placebo			
			2000 IU/d (12 wk)	Placebo			
			100,000 IU bolus, then 4000 IU/d (28 wk)	Placebo			
			800 IU/first 2 mth (6 mth)	Placebo			
			2000 IU//d (15 wk)	Placebo			
			100,000/mth, + ≤ 1000 IU/d (1 yr)	Placebo + 400-1000 IU/d			
Das et al. 2018 [26]	7	n= 1529 participants<br>Both sexes<br>Age: 1 mth – 5 yr	<u>Vitamin D_3</u><br>1000 IU<br>children < 1 yr; 5 d<br>2000 IU<br>children > 1 yr; 5 d	Placebo	Time to resolution of acute pneumonia	MD -0.95 (-6.14, 4.24) (3 studies)	High
					Duration of hospitalisation	MD 0.49 (-8.41, 9.40) (4 studies)	

Meta-analyses of RCTs							
**Author, year**	**Included studies (n)**	**Participants (n), gender, age**	**Vitamin D dose**	**Control/ Comparator**	**Outcome**	**Results/ Summary statistics (95% CI)**	**AMSTAR 2**
			100,000 IU bolus (i.m.; 3 mth)<br><br>100,000 IU bolus (29 mth)<br><br>100,000 IU bolus (2 mth)<br><br>50,000 IU/d for 2 d (12 mth)<br><br>1000 IU children < 1 yr; 5 d<br>2000 IU children > 1 yr; 5 d<br><br>100,000 IU bolus (12 mth)				
Vuichard Gysin et al. 2016 [27]	14	n= 7053 participants<br><br>Both sexes<br>Average age: 19 yr	Vitamin D_3<br>Years 1-2: 800 IU<br>Year 3: 2000 IU + calcium 1200-1500 mg/d (3 yr)<br><br>300 IU/d with mongolian milk (7 wk)<br><br>1000 IU/d (8 wk)<br><br>2000 IU/d (12 wk)	Calcium 1200-1500 mg/d<br><br>Non fortified Milk<br><br>No treatment<br><br>Placebo	Risk of clinical RTI	Vitamin D vs. control (14 studies)<br>RR 0.94 (0.88, 1.00)	High
					Risk of laboratory confirmed RTI	Vitamin D vs. control (4 studies)<br>RR 0.90 (0.68, 1.21)	
					Mean duration of RTI symptoms	Vitamin D vs. control (6 studies)<br>MD -0.06 (-0.29, 0.18)	
					Number of days absent from	Vitamin D vs. control (3 studies)	

Meta-analyses of RCTs							
**Author, year**	**Included studies (n)**	**Participants (n), gender, age**	**Vitamin D dose**	**Control/ Comparator**	**Outcome**	**Results/ Summary statistics (95% CI)**	**AMSTAR 2**
			+/-10,000 IU/wk (8 wk)<br>400 or 800 IU/d (12 mth)<br>400 IU/d (6 mth)<br>2000 IU/d (3 mth)<br>100,000 IU/3 mth (18 mth)<br>Month 0 and 1: 200,000 IU/ mth then 100,000 IU/ mth (18 mth)<br>30,000 or 60,000 IU/ mth (12mth)<br>20,000 IU/wk (17 wk)<br>1200 IU/d (4 mth)<br>2000 IU/d (8 wk)	Placebo<br>Placebo<br>Placebo<br>Placebo<br>Placebo<br>Placebo<br>Placebo +/- calcium<br>Placebo<br>Placebo<br>Placebo	work/school due to RTI<br><br>Severity of RTI	MD 0.06 (-0.41, 0.54)<br><br>Vitamin D vs. control (5 studies)<br>OR 0.95 (0.76, 1.18)	
Yakoob et al. 2016 [28]	2	n= 3134 participants<br><br>Both sexes<br>Age: <12 mth	Vitamin D_3<br>402 IU/d (12 mth)<br>100,000 IU/3 mth (18 mth)	No treatment<br>Placebo	Incidence rate of first or only episode of pneumonia	Rate Ratio 1.06 (0.89, 1.26)	High

Meta-analyses of RCTs							
**Author, year**	**Included studies (n)**	**Participants (n), gender, age**	**Vitamin D dose**	**Control/ Comparator**	**Outcome**	**Results/ Summary statistics (95% CI)**	**AMSTAR 2**
Xiao et al. 2015 [6]	7	n= 6503 participants<br><br>Both sexes<br>Age: < 18 yr	Vitamin D_3<br>1200 IU/d (4 mth)<br><br>100,000 IU bolus (3 mth)<br><br>1400 IU/wk (6 mth)<br><br>500 IU/d (6 mth)<br><br>100,000 IU/3 mth (18 mth)<br><br>300 IU/d (7 wk)<br><br>1000 IU < 1 yr (age)<br>2000 IU > 1 yr (age)<br>(5 d)	NR	Risk of ARI	RR 0.79 (0.55, 1.13) (4 studies)	High
					Repeat episodes of pneumonia	RR 1.16 (0.55, 2.45) (2 studies)	
					Risk of pneumonia	RR 1.06 (0.90, 1.25) (2 studies)	
					Hospital admission due to ARI	RR 0.95 (0.72, 1.26) (2 studies)	
					Influenza A	RR 0.58 (0.34, 1.00) (1 study)	
Bergman et al. 2013 [29]	11	n= 5660 participants<br><br>Both sexes<br>Average age: 16 yr	Vitamin D<br>Frequency: once to every 3 mth<br><br>average daily doses: 800 or 2000 IU (3yr)<br><br>4000 IU (12 mth)<br><br>300 IU (7 wk)<br><br>3344 IU (12 wk)<br><br>400 IU (6 mth)	Placebo	Risk of RTI	Vitamin D vs. control:<br>OR 0.64 (0.49, 0.84)	High

Meta-analyses of RCTs							
**Author, year**	**Included studies (n)**	**Participants (n), gender, age**	**Vitamin D dose**	**Control/ Comparator**	**Outcome**	**Results/ Summary statistics (95% CI)**	**AMSTAR 2**
			2000 IU (3 mth)<br>500 IU (6 mth)<br>100,000 IU (3 mth)<br>1296 IU (18 mth)<br>3653 IU (18 mth)<br>1200 IU (4 mth)				
Mao and Huang 2013 [30]	7	n= 4827 participants<br>Both sexes<br>Age: 1 month-63 yr	Vitamin D<br>2000 IU/d (3 mth)<br>400 IU/d (6 mth)<br>1200 IU/d (4 mth)<br>300 IU/d (1,75 mth)<br>1111-6800 IU/d (6 mth)<br>100,000 IU/3 mth (18 mth)<br>200,000 IU/mth (2 mth), then 100,000 IU/mth (18 mth)	Placebo	Risk of RTI	RR 0.98 (0.93, 1.03)	High
Charan et al. 2012 [31]	5	n= 943 participants	Vitamin D<br>400 IU/d (6 mth)	Placebo	Incidence of RTI	Vitamin D vs. control (5 studies)<br>OR 0.582 (0.417, 0.812)	Moderate

Meta-analyses of RCTs							
**Author, year**	**Included studies (n)**	**Participants (n), gender, age**	**Vitamin D dose**	**Control/ Comparator**	**Outcome**	**Results/ Summary statistics (95% CI)**	**AMSTAR 2**
		Both sexes<br>Age: 1 to 15 and ≥ 18 yr	1200 IU/d (4 mth)<br>1200 IU/d (3 mth)<br>100,000 IU bolus (3 mth)<br>2000 UI/d (3 yr)			Vitamin D vs. control in adult population (3 studies):<br>OR 0.544 (0.278, 1.063)	
						Vitamin D vs. control in paediatric population (2 studies):<br>OR 0.579 (0.416, 0.805)	

Table S11: Meta-analyses of prospective cohort studies – ARI

Meta-analyses of prospective cohort studies							
**Author, year**	**Included studies (n)**	**Participants (n), gender, age**	**Vitamin D dose**	**Control/ Comparator**	**Outcome***	**Results/ Summary statistics (95% CI)**	**AMSTAR 2**
Pacheco-Gonzalez et al. 2018 [10]	13	n= 8370 mother-child-pairs<br>Both sexes<br>Age: 0-3 yr	25(OH)D levels in maternal blood or cord blood	-	Risk of RTI	Highest vs. lowest 25(OH)D<br>OR 0.64 (0.47, 0.87)	High
Feng et al. 2017 [12]	10	n= 8359 mother-child-pairs<br>Both sexes<br>Age: NR	25(OH)D levels in maternal blood or cord blood	-	Risk of RTI	Highest vs lowest 25(OH)D (9 studies):<br>OR 0.85 (0.66, 1.09)	High
						Per 10 nmol/l increment in 25(OH)D (9 studies):<br>OR 0.97 (0.94, 1.01)	

* Outcome variable as described in the respective systematic review

Table S12: Systematic Reviews of RCTs – ARI

**Systematic Reviews of RCTs**							
**Author, year**	**Included studies (n)**	**Participants (n), gender, age**	**Vitamin D dose**	**Control/ Comparator**	**Outcome***	**Results**	**AMSTAR 2**
Autier et al. 2014 [24]	5	n= 6057 participants<br><br>Both sexes<br>Age: NR	Vitamin D<br>range: 800-400 IU/d (3-62 mth)		URTI<br>(11 outcomes were assessed by trials)	Overall 2 outcomes with significant improvements	Low
Das et al. 2013 [32]	2	n= 653 participants<br><br>Both sexes<br>Age: 1 mth- 5 yr	Vitamin D_3<br>100,000 IU bolus (duration: NR)<br><br>1000 IU < 1 yr (age)<br>2000 IU > 1 yr (age)<br>(5 d)	Placebo	Time period to resolution or recovery from pneumonia	No beneficial effect of vitamin D supplementation in acute (severe and non-severe) pneumonia.	Moderate
Jolliffe et al. 2013 [33]	14	n= 11,431 participants<br><br>Both sexes<br><br>Age: NR, infants, children and adults	Vitamin D_3<br>800 IU/d for 2 years, then 2,000 IU/d for 1 year (3 yr)<br><br>800 IU/d alone or 800 IU/d + calcium (2 yr)<br><br>2000 IU/d (3 mth)<br><br>2000 IU/d (1 yr)<br><br>1200 IU/d (4 mth)<br><br>400 IU/d (6 mth)<br><br>100,000 IU single bolus (3 mth)	Placebo<br><br>Placebo<br><br>Placebo<br><br>800 IU/d<br><br>NR<br><br>Placebo<br><br>Placebo	Risk of ARI	Vitamin D supplementation protected against ARI<br>(7 studies) – in the study population as a whole<br>(6 studies) and in a subgroup with profound vitamin D deficiency (1 study)<br>Null effects for all respiratory outcomes investigated<br>(6 studies).<br><br>Null effect of vitamin D supplementation on primary outcome (pneumonia incidence) with a negative effect on one secondary	Very low

Systematic Reviews of RCTs							
**Author, year**	**Included studies (n)**	**Participants (n), gender, age**	**Vitamin D dose**	**Control/ Comparator**	**Outcome***	**Results**	**AMSTAR 2**
			1400 IU/wk (6 mth)<br>500 IU/d (6 mth)<br>100,000 IU/mth (1 yr)<br>100,000 IU/3 mth (18 mth)<br>1111-6800 IU/d (6 mth)<br>300 IU/d (7 wk)<br>month 1 & 2: 200,000 IU bolus, then 100,000 IU/mth (18 mth)	Placebo<br>Placebo<br>Placebo<br>Placebo<br>Placebo<br>Placebo<br>Placebo		outcome (vitamin D increased incidence of repeat episodes of radiologically confirmed pneumonia) (1 study)	

* Outcome variable as described in the respective systematic review

Table S13: Systematic Reviews of prospective cohort studies – ARI

Systematic Reviews of prospective cohort studies							
**Author, year**	**Included studies (n)**	**Participants (n), gender, age**	**Vitamin D dose**	**Control/ Comparator**	**Outcome***	**Results**	**AMSTAR 2**
Fried et al. 2016 [34]	12	n= 8822 mother-child pairs<br>Both sexes<br>Age: 0-3 yr	25(OH)D levels in maternal blood or cord blood	-	Risk of RTI	LRTI:<br>Significant protective associations between 25(OH)D (6 studies) and LRTI (or URTI (1 study)).<br>Increased ORs of LRTI in children born to mothers with higher 25(OH)D (2 studies).	Moderate

Systematic Reviews of prospective cohort studies							
**Author, year**	**Included studies (n)**	**Participants (n), gender, age**	**Vitamin D dose**	**Control/ Comparator**	**Outcome***	**Results**	**AMSTAR 2**
						No association between 25(OH)D levels and LRTI (3 studies).<br><br>Other RTIs:<br>No association between maternal 25(OH)D levels and other RTIs (2 studies).<br>No association with otitis media (2 studies), increased ORs of any RTI in children with lower maternal 25(OH)D levels (1 study).<br>More recurrent RTIs in children born to vitamin D-deficient mothers (1 study).	
Autier et al. 2014 [24]	3	n= 7787 participants<br><br>Both sexes<br>Age: NR	25(OH)D	-	Risk of RTI	RR for highest vs. lowest 25(OH)D: 0.50 to 0.95 (1 study)<br>Inverse association between RTI risk and 25(OH)D levels (outcome as a continuous variable) (2 studies)	Low
					Days of absence due to RTI	RR for highest vs. lowest 25(OH)D: 0.50 to 0.95 (1 study)	
Jolliffe et al. 2013 [33]	11 (3 birth cohort studies)	n= 6627<br><br>Both sexes	25(OH)D (serum or maternal blood or cord blood)	-	Risk of ARI	Inverse associations between low serum 25(OH)D and risk of ARI (7 studies).	Very low

Systematic Reviews of prospective cohort studies							
**Author, year**	**Included studies (n)**	**Participants (n), gender, age**	**Vitamin D dose**	**Control/ Comparator**	**Outcome***	**Results**	**AMSTAR 2**
		Age: NR; Infants, children and adults				Serum 1,25(OH)$_2$D levels may be protective (as evidenced by higher serum 1,25(OH)$_2$D levels or by administration of 1-alpha-hydroxylated vitamin D metabolites) (2 studies).<br><br>No association (3 studies) and a positive association (1 study) between higher maternal serum 25(OH)D levels in late pregnancy and increased risk of LRTI in offspring during infancy.	

* Outcome variable as described in the respective systematic review

Table S14: Meta-analyses of RCTs – Cognitive decline

Meta-analyses of RCTs							
**Author, year**	**Included studies (n)**	**Participants (n), gender, age**	**Vitamin D dose**	**Control/ Comparator**	**Outcome**	**Results/ Summary statistics (95% CI)**	**AMSTAR 2**
Goodwill et al. 2017 [35]	2 RCTs and 1 retro-spective pre-post study	n=314<br><br>Sex: NR<br>Age: ≥ 18 yr (two trials with elderly participants)	Vitamin D_3<br>5000 IU Vitamin D_3/d (6 wk)<br><br>Vitamin D_2<br>3x50,000 IU/week (4 wk)	Placebo	Cognition	SMD 0.21 (-0.05, 0.46)	High

Meta-analyses of RCTs							
**Author, year**	**Included studies (n)**	**Participants (n), gender, age**	**Vitamin D dose**	**Control/ Comparator**	**Outcome**	**Results/ Summary statistics (95% CI)**	**AMSTAR 2**
			600,000 IU (single injection) (6 mth)				
Annweiler et al. 2013 [36]	3 (2 open pre-post study designs, 1 double-blind RCTs)	n=234<br><br>Sex: NR<br>Age: ≥ 18 yr	Vitamin D_3<br>800 or 100,000 IU/mth (7.8 mth)<br><br>5000 IU/d (6 wk)<br><br>Vitamin D_2<br>50,000 IU x 3/week (4 wk)	No vitamin D supplements<br><br>Placebo<br><br>NR	impaired executive functions before and after vitamin D supplementation	Effect size -0.50 (-0.69, -0.32)	Moderate
					impaired executive functions at the end of follow-up	Effect size 0.14 (-0.04, 0.32)	

Table S15: Meta-analyses of prospective cohort studies – Dementia and cognitive decline

Meta-analyses of prospective cohort studies							
**Author, year**	**Included studies (n)**	**Participants (n), gender, age**	**Vitamin D dose**	**Control/ Comparator**	**Outcome**	**Results/ Summary statistics (95% CI)**	**AMSTAR 2**
Chen et al. 2018 [37]	10	n=28,640<br><br>Both sexes<br>Mean age: 56-84.6 yr	25(OH)D level		Dementia<br>Alzheimer´s disease	Dementia (10 studies); highest vs. lowest: RR 0.72 (0.59, 0.88)	High
						Dose response analysis (7 studies); risk of dementia for every 10 nmol/l increment in 25(OH)D: RR 0.95 (0.93, 0.98)<br><br>p for nonlinearity = 0.176 (non-significant)	
						Alzheimer's disease (6 studies); highest vs. lowest: RR 0.78 (0.60, 1.00)	

Meta-analyses of prospective cohort studies							
**Author, year**	**Included studies (n)**	**Participants (n), gender, age**	**Vitamin D dose**	**Control/ Comparator**	**Outcome**	**Results/ Summary statistics (95% CI)**	**AMSTAR 2**
						Dose response analysis (4 studies); risk of Alzheimer's disease for every 10 nmol/l increment in 25(OH)D: RR 0.93 (0.89, 0.97)<br>p for nonlinearity = 0.804 (non-significant)	
Jayedi et al. 2018 [38]	8 cohorts from 1966- 2017 (seven pro-spective and one retro-spective cohort study)	Dementia: n=18,168 (1953 cases)<br>Alzheimer´s disease: n= 25,520 (1607 cases)<br>Both sexes Age: ≥ 18 yr	25(OH)D level<br>sufficiency: ≥ 50 nmol/l insufficiency: 25-50 nmol/l<br>deficiency: <25 nmol/l		Dementia Alzheimer´s disease	Vitamin D insufficiency (6 studies) and dementia: pooled HR 1.09 (0.95, 1.24)	High
						Vitamin D deficiency (5 studies) and dementia: pooled HR 1.33 (1.08, 1.58)	
						Risk of dementia for a 25-nmol/l increment in serum 25(OH)D (7 studies): pooled HR 0.83 (0.70, 0.96)	
						Nonlinear dose-response analysis: U-shaped association with a nadir at ~62 nmol/l serum 25(OH)D<br>Risk of dementia decreased continuously with increasing serum levels of 25(OH)D from a baseline of ~13 nmol/l up to ~80 nmol/l (after excluding the study reporting risk estimates	

Meta-analyses of prospective cohort studies							
**Author, year**	**Included studies (n)**	**Participants (n), gender, age**	**Vitamin D dose**	**Control/ Comparator**	**Outcome**	**Results/ Summary statistics (95% CI)**	**AMSTAR 2**
						of dementia for serum 25(OH)D levels > ~88 nmol/l	
						Vitamin D insufficiency and Alzheimer's disease (4 studies): pooled HR 1.19 (0.96, 1.41)	
						Vitamin D deficiency and Alzheimer's disease (3 studies) HR 1.31 (0.98, 1.65)	
						risk of Alzheimer´s disease for a 25-nmol/l increment of serum 25(OH)D (6 studies): pooled HR 0.83 (CI 0.68, 0.98)	
						nonlinear dose-response analyses: continuous decrement in risk with increasing serum 25(OH)D levels from a baseline of ~13 nmol/l up to ~ 88nmol/l	
Goodwill et al. 2017 [35]	14	n~30,000/ NR<br><br>Both sexes<br>Age: ≥ 18 yr (in the majority of the included studies participants were >40 yr)	High vs. low 25(OH)D level<br><br>(no thresholds available)	-	Cognition	Low vitamin D and cognitive decline:<br>OR 1.14 (1.06, 1.23)	High

Meta-analyses of prospective cohort studies							
**Author, year**	**Included studies (n)**	**Participants (n), gender, age**	**Vitamin D dose**	**Control/ Comparator**	**Outcome**	**Results/ Summary statistics (95% CI)**	**AMSTAR 2**
Sommer et al. 2017 [39]	5 (4 pro-spective studies, 1 retro-spective study)	n=18,933<br>Both sexes Adults	25(OH)D level<br>no deficiency or sufficient supply: ≥ 50 nmol/l<br>insufficiency: ≥ 25 to <50 nmol/l<br>serious deficiency: < 25 nmol/l	-	Dementia	Serious deficiency (< 25 nmol/l or 7-28 nmol/l) vs. sufficient supply (≥ 50 nmol/l or 54-159 nmol/l)<br>Point estimate: 1.54 (1.19, 1.99)<br>Vitamin D deficiency increased the risk of dementia.	High
Cao et al. 2016 [40]	3	n=12,702<br>Both sexes Age: ≥ 20 yr	Vitamin D status 25(OH)D	-	Dementia, mild cognitive impairment	Low levels of vitamin D and cognitive decline: RR 1.52 (1.17, 1.98)	Very low
Shen and Ji 2015 [41]	2	n = 8086<br>Both sexes Average Age: 73.6 yr/NR	Vitamin D status (deficiency: ≤ 50 nmol/l)<br>≤ 50 vs. > 50 nmol/l	-	Alzheimer´s disease Dementia	Risk in vitamin D deficient subjects: Alzheimer's disease risk (n=2): OR 1.21 (1.01, 1.40)<br>Dementia risk (n=1)*: OR 1.63 (1.09, 2.16)<br>*the results including 3 additional cross-sectional studies did not differ: OR 1.49 (1.09, 1.88)	Low

Meta-analyses of prospective cohort studies							
**Author, year**	**Included studies (n)**	**Participants (n), gender, age**	**Vitamin D dose**	**Control/ Comparator**	**Outcome**	**Results/ Summary statistics (95% CI)**	**AMSTAR 2**
Annweiler et al. 2013 [36]	3	n=4095/NR<br><br>Both sexes<br>Mean age: ~75 yr	Vitamin D status (higher vs. lower 25(OH)D concentrations)	-	Cognitive (executive) function<br><br>Executive function refer to a hetero-geneous set of high-level processes that control and regulate other abilities and behaviours	Risk of incident decline of TMT-B score:<br>OR 1.25 (1.05, 1.48)<br><br>Participants with lower 25(OH)D concentrations had a 1.25 times greater risk of worsening TMT-B score in longitudinal follow-ups compared to those with higher 25(OH)D concentrations, indicating that low vitamin D status may precede decline of executive functions	Moderate
Etgen et al. 2012 [42]	2	n=497/90<br>Sex: NR<br>Age:≥ 65 yr	Vitamin D deficiency vs. normal vitamin D concentrations	-	Cognitive impairment	OR 2.49 (1.74, 3.56)	Low

Table S16: Systematic Reviews of RCTs – Cognitive decline

Systematic Reviews of RCTs							
**Author, year**	**Included studies (n)**	**Participants (n), gender, age**	**Vitamin D dose**	**Control/ Comparator**	**Outcome**	**Results**	**AMSTAR 2**
Lerner et al. 2018 [43]	3	n=222<br><br>Men and women<br>Age: NR	Vitamin D<br>dosage: NR	Placebo/NR	Cognitive impairment	Vitamin D_3 supplements were associated with medium-term improvement in cognitive performance in older adults	Low

Systematic Reviews of RCTs							
**Author, year**	**Included studies (n)**	**Participants (n), gender, age**	**Vitamin D dose**	**Control/ Comparator**	**Outcome**	**Results**	**AMSTAR 2**
						and in particular with better executive functioning<br><br>Younger adults: Vitamin D status may be important for both executive functioning and mental health (1 study). No effect of vitamin D supplements on cognitive or emotional functioning. (1 study)	
Autier et al. 2014 [24]	1	n=4143<br><br>sex: NR<br>Age: elderly	Vitamin D<br>400 IU/d (84 mth)	NR	Dementia, mild cognitive impairment	No significant differences in incident dementia or mild cognitive impairment, or in global or domain-specific cognitive function.	Low
Balion et al. 2012 [44]	3	n=354<br><br>Both sexes<br>Age: 74-87 yr	Vitamin D<br>Oral supplement containing various nutrients including 160 IU/d (12 mth)<br><br>Nutrient dense drink containing 520 IU/d (24 wk)<br><br>Vitamin D_2<br>9000 IU/d (8–40 wk)	Placebo containing calcium and magnesium<br><br>Placebo drink containing no vitamins or minerals<br><br>Placebo	Cognitive function	No significant differences between treatment and control group for almost all cognitive tests (2 studies).<br><br>Significant differences between treatment and control groups for almost all cognitive tests (except long-term memory recall test) (1 study).	High

Systematic Reviews of RCTs							
**Author, year**	**Included studies (n)**	**Participants (n), gender, age**	**Vitamin D dose**	**Control/ Comparator**	**Outcome**	**Results**	**AMSTAR 2**
Etgen et al. 2012 [42]	2	n = 202<br><br>both sexes elderly persons	Vitamin D_2<br>600,000 IU (single injection)<br><br>50,000 IU x 3/wk (4 wk)	Placebo<br><br>No active medical intervention	Cognitive function	Vitamin D_2 led to a significant improvement of choice reaction time compared with placebo (1 study).<br>Neurocognitive performance did not improve significantly (1 study).	Low

Table S17: Systematic Reviews of prospective cohort studies – Dementia and cognitive decline

Systematic Reviews of prospective cohort studies							
**Author, year**	**Included studies (n)**	**Participants (n), gender, age**	**Vitamin D dose**	**Control/ Comparator**	**Outcome**	**Results**	**AMSTAR 2**
Lerner et al. 2018 [43]	6 cohort	n=11,981<br><br>Both sexes<br>Age: NR	Vitamin D status	-	Cognitive impairment	Vitamin D deficiency was associated with cognitive impairment (4/6 studies; 3 studies included elderly participants).<br><br>Vitamin D_3 supplements were associated with medium-term improvement in cognitive performance in older adults and in particular with better executive functioning.<br><br>In younger adults vitamin D status may be important for both executive functioning and	Low

Systematic Reviews of prospective cohort studies							
**Author, year**	**Included studies (n)**	**Participants (n), gender, age**	**Vitamin D dose**	**Control/ Comparator**	**Outcome**	**Results**	**AMSTAR 2**
						mental health (1 study). Another study showed no effect of vitamin D supplementation on cognitive or emotional functioning (1 study).	
Killin et al. 2016 [45]	3	n=11,884 (691 cases)<br><br>Both sexes<br>Age: NR	Vitamin D status	-	Dementia	Lower vitamin D levels at baseline were associated with an increased risk of developing dementia.<br><br>Overall strength of evidence: strong evidence = there is a reported association with dementia in the majority of published papers	Low
Autier et al. 2014 [24]	5	Cognitive function: n=10,358 (260 cases)<br><br>Non-Alzheimer disease: n=40 (6 cases)<br><br>Sex: NR<br>Age: elderly	Highest vs. lowest 25(OH)D	-	Cognitive function (4 studies), Non-Alzheimer dementia (1 study)	<u>Cognitive function:</u> Decreasing risk for reduced cognitive function with higher 25(OH)D (RR of highest vs. lowest quintile 0.50 to 0.95) (3 studies; 2 of them significant).<br><br>Inverse relation between serum 25(OH)D concentrations (1 study; multiple linear regression).<br><br><u>Non-Alzheimer disease:</u>	Low

Systematic Reviews of prospective cohort studies							
**Author, year**	**Included studies (n)**	**Participants (n), gender, age**	**Vitamin D dose**	**Control/ Comparator**	**Outcome**	**Results**	**AMSTAR 2**
						Significantly decreasing risk (RR of highest vs. lowest quintile < 0.50) for Non-Alzheimer dementia with higher 25(OH)D levels (1 study).	
van der Schaft et al. 2013 [46]	6	n=10,896<br><br>Both sexes<br>Age: ≥ 65 years	Serum 25(OH)D concentration	-	Cognitive function	Statistically significant decline on ≥1 cognitive function test or higher frequency of dementia in participants with lower vitamin D levels or intake compared to participants with higher vitamin D levels or intake (4/6 studies).	High
Balion et al. 2012 [44]	2	n= 2464<br><br>Both sexes (one study without women)<br>Age:≥ 65 yr	Vitamin D status	-	Cognitive function	No significant association between vitamin D quartile and baseline cognitive impairment or incident cognitive decline (1 study).<br><br>Participants deficient in 25(OH)D (25 nmol/l) experienced an increased risk of substantial cognitive decline over 6 years, compared to those with sufficient concentrations (75 nmol/l). Individuals with 25(OH)D concentrations 25 nmol/l declined by an	High

Systematic Reviews of prospective cohort studies							
**Author, year**	**Included studies (n)**	**Participants (n), gender, age**	**Vitamin D dose**	**Control/ Comparator**	**Outcome**	**Results**	**AMSTAR 2**
						additional 0.3 points per year compared to those sufficient in 25(OH)D (75 nmol/l), even after restricting the sample to individuals without dementia (1 study).	

Table S18: Meta-analyses of RCTs – Depression

Meta-analyses of RCTs							
**Author, year**	**Included studies (n)**	**Participants (n), gender, age**	**Vitamin D dose**	**Control/ Comparator**	**Outcome**	**Results/ Summary statistics (95% CI)**	**AMSTAR 2**
Vellekkatt and Menon 2018 [47]	4	n=948<br><br>Sex and age: NR	Vitamin D_3<br>50,000 IU/wk (52 wk)<br><br>50,000 IU/wk (8 wk)<br><br>300,000/150,000 IU (i.m. single dose; 12 wk)<br><br>1500 IU + 20 mg Fluoxetine/d (8 wk)	 Placebo<br><br>Placebo<br><br>No treatment<br><br><br>Fluoxetine	Depressive symptoms	Effect size: 0.58 (0.45, 0.72)	Moderate
Gowda et al. 2015 [48]	9	n=4923<br><br>Both sexes<br>Age: ≥ 18 yr	Vitamin D<br>20,000 or 40,000 IU/d (1yr)<br><br>50,000 IU/wk (8 wk)<br><br>5000 IU/d (6 wk)	Placebo	Depressive symptoms	SMD 0.28 (-0.14, 0.69)	High

Meta-analyses of RCTs							
**Author, year**	**Included studies (n)**	**Participants (n), gender, age**	**Vitamin D dose**	**Control/ Comparator**	**Outcome**	**Results/ Summary statistics (95% CI)**	**AMSTAR 2**
			400 IU/d (+calcium + antidepressants; 2yr)<br><br>40,000 IU/wk (6 mth)<br><br>Vitamin D_3<br>400 or 800 IU/d (5d)<br><br>500,000 IU/y (3-5 yr)<br><br>1500 IU/d (8 wk)<br><br>Calcitriol<br>2,000,000 IU x 2/d (36 mth)				
Spedding 2014 [49]	4	n=4610<br><br>Both sexes<br>Age: NR	Vitamin D<br>range: 400-18,400 IU/d	Placebo or NR	Depression symptoms	Studies were grouped according to the presence of biological flaws (e.g. 25(OH)D not assessed, dose not appropriate):<br><br>Studies without flaws<br>Meta-analysis (2 studies):<br>SMD 0.78 (0.24, 1.27)<br>Studies with flaws<br>Meta-Analysis (2 studies):<br>SMD -1.1 (-0.7, -1.5)	Low
Shaffer et al. 2014 [50]	7	n=3191<br><br>Both sexes<br>Age: 18-79 yr	Vitamin D_3<br>600 IU/d (8 wk)	Placebo	Depressive symptoms	SMD -0.14 (-0.33, 0.05)	High

Meta-analyses of RCTs							
**Author, year**	**Included studies (n)**	**Participants (n), gender, age**	**Vitamin D dose**	**Control/ Comparator**	**Outcome**	**Results/ Summary statistics (95% CI)**	**AMSTAR 2**
			20,000 or 40,000 IU/wk (+ calcium; 1 yr)<br>5000 IU/d (6wk)<br>400 IU/d (+ calcium; 1 yr)<br>20,000 IU/wk (6 mth)<br>1500 IU/d + fluoxetine (8 wk)<br>150,000 or 300,000 IU IM injection	Placebo<br>Placebo<br>Placebo<br>Placebo<br>Placebo + fluoxetine<br>no injection			
Li et al. 2014 [51]	6	n=1203<br>71 depressed patients<br>72% females<br>Age: NR	Vitamin D_3<br>20,000 or 40,000 IU/wk (12 mth)<br>50,000 IU/wk (8 wk)<br>500,000 IU/yr (bolus) (3-5 yr)<br>20,000 IU/wk (6 mth)<br>1500 IU/d + fluoxetine (8 wk)<br>Calcitriol<br>10 IU/twice a day (3 yr)	Placebo<br>Placebo<br>Placebo<br>Placebo<br>Placebo + fluoxetine<br>Placebo	Depression	Postintervention SMD of depression scores:<br>-0.14 (-0.41, 0.13)<br>OR of depression for vitamin D supplementation vs. placebo (2 studies):<br>0.93 (0.54, 1.59)	High

Table S19: Meta-analyses of prospective cohort studies – Depression

Meta-analyses of prospective cohort studies							
**Author, year**	**Included studies (n)**	**Participants (n), gender, age**	**Vitamin D dose**	**Control/ Comparator**	**Outcome**	**Results/ Summary statistics (95% CI)**	**AMSTAR 2**
Wang et al. 2018 [52]	3	n=4593<br><br>Women<br>Age: NR	Vitamin D status<br><br>Vitamin D deficiency: 25(OH)D <30 nmol/l	-	Antepartum depression	OR 1.47 (0.92, 2.35)	High
	4	n=2228<br><br>Women<br>Age: NR	Vitamin D status<br><br>Vitamin D deficiency: 25(OH)D <50 nmol/l	-	Postpartum depression	OR 3.67 (1.72, 7.85)	High
Ju et al. 2013 [53]	4	n=12,648 (2663 cases)<br>Both sexes<br>Age: ≥40 yr	Vitamin D status	-	Depression	10 ng/ml increase in 25(OH)D levels: OR 0.92 (0.87, 0.98)	Moderate
						15 ng/ml increase in 25(OH)D levels: OR 0.88 (0.81, 0.96)	
						20 ng/ml increase in 25(OH)D levels: OR 0.85 (0.76, 0.95)	
Anglin et al. 2013 [54]	3	n=8815<br><br>Both sexes<br>Age: ≥50 yr	Vitamin D status	-	Depression	Lowest vs. highest vitamin D status: HR 2.21 (1.40, 3.49)	Moderate
						Change in the ln(HR) of depression per 20 nmol(L change in vitamin D level:<br>β = -0.19 (-0.41, 0.04)	
						Vitamin D deficiency using cut-off points of 50 nmol/l and 37.5 nmol/l: HR 1.04 (0.59, 1.86)<br><br>The HRs of depression for those with and without vitamin D levels below 50 nmol/l	

Meta-analyses of prospective cohort studies							
**Author, year**	**Included studies (n)**	**Participants (n), gender, age**	**Vitamin D dose**	**Control/ Comparator**	**Outcome**	**Results/ Summary statistics (95% CI)**	**AMSTAR 2**
						(2 studies) were pooled with the HR of depression for vitamin D below vs. above 37.5 nmol/l (1 study).	
						Vitamin D deficiency using cut-off points of 50 nmol/l and 75 nmol/l: HR 1.31 (0.97, 1.77)	

Table S20: Systematic Reviews of RCTs– Depression

Systematic Reviews of RCTs							
**Author, year**	**Included studies (n)**	**Participants (n), gender, age**	**Vitamin D dose**	**Control/ Comparator**	**Outcome**	**Results**	**AMSTAR 2**
Aghajafari et al. 2018 [55]	2 (secondary analysis of 1 RCT)	n=279<br><br>Women<br>Age: NR	Vitamin D<br>2000 IU/d from 26-28 week to birth<br><br>1 study:<br>NR	Placebo<br><br>for 1 study:<br>NR	Antenatal depression	Low vitamin D levels in early pregnancy were associated with higher depressive symptom scores in early and late pregnancy.<br>Significant association between lower levels of vitamin D and antenatal depression (1 study).	High
	3 (secondary analyses of 2 RCTs)	n=1319<br><br>Women<br>Age: NR	Vitamin D<br>2000 IU/d from 26-28 week to birth<br><br>NR (2 studies)	Placebo<br><br>NR (2 studies)	Postpartum depression	No association (2 studies); vitamin D supplementation was effective in decreasing postpartum depression levels (1 study).	High

Systematic Reviews of RCTs							
**Author, year**	**Included studies (n)**	**Participants (n), gender, age**	**Vitamin D dose**	**Control/ Comparator**	**Outcome**	**Results**	**AMSTAR 2**
Lerner et al. 2018 [43]	2	n=2159<br><br>Both sexes<br>Age: NR	Vitamin D_3<br>1500 IU/d + fluoxetine (8 wk)<br><br>Vitamin D<br>800 IU/d (6 mth)	Placebo + fluoxetine<br><br>NR	Depression	Vitamin D + Fluoxetine combination was superior to fluoxetine alone in controlling depressive symptoms (1 study). Vitamin D supplementation did not lead to an improvement in mental health scores (1 study).	Low
Föcker et al. 2017 [56]	21	n=43,340<br><br>Both sexes<br>Age: adults	Vitamin D<br>400 IU/d + 377 mg calcium/d (1 yr)<br><br>400 or 800 IU/d (5 d)<br><br>100,000 IU once<br><br>4000 IU/wk (1 yr)<br><br>800 IU/d + 1000 mg calcium/d (6 mth)<br><br>20,000 or 40,000 IU/wk (1 yr)<br><br>8,000,000 IU/d + 500 mg calcium/d (2 menstrual cycles)<br><br>50,000 IU/wk (8 wk)<br><br>9200 IU/d (8 wk) + calcium	377 mg calcium/d<br><br>0 IU/d<br><br>Photo-therapy/mth<br><br>600 IU/d<br><br>No treatment<br><br>Placebo<br><br>Placebo<br><br>Placebo<br><br>No treatment	Mental health (Mood, Depression, Seasonal affective symptoms, Fibromyalgia, Wellbeing, PMS)	No effect on mental health (11 studies); beneficial effect on parameters of mental health (10 studies).	Very low

Systematic Reviews of RCTs							
**Author, year**	**Included studies (n)**	**Participants (n), gender, age**	**Vitamin D dose**	**Control/ Comparator**	**Outcome**	**Results**	**AMSTAR 2**
			5000 IU/d (6 wk)	Placebo			
			500,000 IU once (3-5 yr)	Placebo			
			2000 IU/d (8 d)	500 mg vitamin C			
			400 IU/d + 1000mg calcium (3 yr)	Placebo			
			20,000,000 IU/d (3 yr) or 20,000,000 IU/d+ hormone therapy	hormone therapy or placebo			
			40,000 IU/wk (6 mth)	Placebo			
			1500 IU/d + 20 mg fluoxetine	20 mg fluoxetine			
			150,000 or 300,000 IU single injection (3 mth)	injection			
			5000 IU/d (8.1-10 d)	2x 500 mg vitamin C/d			
			2800 IU/d (12 wk)	Placebo			
			200,000 IU then 25,000 IU/2 wk (4 mth)	Placebo			
			2000 IU/d (from 26-28 wk of gestation until childbirth)	Placebo			

Systematic Reviews of RCTs							
**Author, year**	**Included studies (n)**	**Participants (n), gender, age**	**Vitamin D dose**	**Control/ Comparator**	**Outcome**	**Results**	**AMSTAR 2**
Autier et al. 2017 [23]	5	n=1111<br><br>Sex and age: NR	Vitamin D<br>very high doses<br>(2-12 mth)	NR	Mood disorders	No effect (4 studies);<br>vitamin D supplementation reduced mood disorders significantly (1 study)	Low
Sarris et al. 2016 [57]	2 (open label CT, double blind RCT)	n=81<br><br>Sex and age: NR	Vitamin D_3<br>1500 IU/d (8 wk)<br><br>300,000 IU once (4 wk)	Placebo<br><br>antidepressant only	Depressive symptoms	Statistically significant reduction in depression rating scores in the treatment group compared with the control group in both studies between baseline and endpoint/over the course of the study	Low
Spedding 2014 [49]	15	n=42,258<br><br>Both sexes<br>Age: NR	Vitamin D<br>400 - 18,400 IU/d<br>(doses not precisely specified: were depicted in bar graph; duration: NR)	NR	Depression symptoms	Studies were grouped according to the presence of biological flaws (e.g. 25(OH)D not assessed, dose not appropriate, high baseline 25(OH)D levels)<br>These flaws limit the ability of these studies to demonstrate a change in vitamin D status in the intervention group.<br><br>Studies without flaws:<br>6/7 studies showed improvement in depression symptoms.<br><br>Studies with flaws:	Low

Systematic Reviews of RCTs							
**Author, year**	**Included studies (n)**	**Participants (n), gender, age**	**Vitamin D dose**	**Control/ Comparator**	**Outcome**	**Results**	**AMSTAR 2**
						6/9 studies showed no effect on depression symptoms.	
Autier et al. 2014 [24]	7	n=7191<br><br>Sex and age: NR	Vitamin D<br>range: 400-5720 IU/d (0.2-60 mth)	NR	Mood disorders (depression)	No effect (5 studies);<br>vitamin D supplementation reduced mood disorders significantly (2 studies)	Low

Table S21: Systematic Reviews of prospective cohort studies – Depression

Systematic Reviews of prospective cohort studies							
**Author, year**	**Included studies (n)**	**Participants (n), gender, age**	**Vitamin D dose**	**Control/ Comparator**	**Outcome**	**Results**	**AMSTAR 2**
Aghajafari et al. 2018 [55]	2	n=4592<br><br>Women<br>Age: NR	Vitamin D status	-	Antenatal depression	Significant association between lower levels of vitamin D and antenatal depression.	High
	4	n=1455<br><br>women<br>age: NR	Vitamin D status	-	Postpartum depression	Lower vitamin D concentration was associated with increased and higher levels of vitamin D were associated with decreased odds of PPD as well as reduced symptoms (3 studies). No association (1 study).	High
Trujillo et al. 2018 [58]	2	n=4279<br><br>Women<br>Age:<br>26.7-31 yr	Vitamin D status	-	Antenatal depression	Serum vitamin D deficiency and insufficiency were significantly associated with an increased likelihood of depression (2 studies).	High

Systematic Reviews of prospective cohort studies							
**Author, year**	**Included studies (n)**	**Participants (n), gender, age**	**Vitamin D dose**	**Control/ Comparator**	**Outcome**	**Results**	**AMSTAR 2**
						1-unit increase of log serum vitamin D levels was significantly associated with a 46% decreased likelihood of depression (1 study).	
	4	n=1441<br><br>Women<br>Age: 26-31 yr	Vitamin D status	-	Postpartum depression	Significant inverse association between serum vitamin D levels and depression scores (3 studies). No association (1 study).	High
Amini et al. 2018 [59]	6	n=2416<br><br>Women<br>Age: NR	Vitamin D status	-	Postpartum depression	In all studies low 25(OH)D was associated with reduced depressive symptoms	Low
Lerner et al. 2018 [43]	3	n=~ 5600<br><br>Both sexes (children and adults)<br>Age: ≤10-65 yr	Vitamin D status	-	Depression	Low vitamin D levels were associated with presence and severity of depression (3 studies).<br>Significant association between low serum vitamin D measured at age 9.8 years and higher scores on depressive symptoms assessed at age 13.8 years but not at age 10.6 years (1 study)	Low
Sparling et al. 2017 [60]	8	n=6705<br><br>Women<br>Age: NR	Vitamin D status	-	Postpartum depression	Protective associations and linear trends between vitamin D concentrations and depression (6/8 studies). No	High

Systematic Reviews of prospective cohort studies							
**Author, year**	**Included studies (n)**	**Participants (n), gender, age**	**Vitamin D dose**	**Control/ Comparator**	**Outcome**	**Results**	**AMSTAR 2**
						significant protective association (2/8 studies).	
Autier et al. 2014 [24]	5	n=6016<br>514 cases<br><br>Sex and age: NR	Vitamin D status	-	Mood disorders (depression)	Highest vs. lowest 25(OH)D: increased frequency of mood disorders associated with low 25(OH)D (4 studies)<br><br>Significant inverse association with mood disorders (1 study; outcome as a continuous variable)	Low

Table S22: Meta-analyses of RCTs – MS

Meta-analyses of RCTs							
Author, year	**Included studies (n)**	**Participants (n), gender, age**	**Vitamin D dose**	**Control/ Comparator**	**Outcome**	**Results/ Summary statistics (95% CI)**	**AMSTAR 2**
Mc Laughlin et al. 2018 [61]	12	n= 950 participants with RRMS or CIS (111 with CIS)<br><br>Both sexes<br>Age: ≥ 15 yr	Vitamin D$_2$<br>6000 IU/d for 2 wk, then adjusted dose (target 25(OH)D levels of 130–175 nmol/l) (6 mth)<br><br>Vitamin D$_3$<br>4370 IU/d (12 mth)<br><br>10,400 IU/d (6 mth)<br><br>2857 IU/d (24 mth)	1000 IU/d<br><br><br><br><br>800 IU/d<br><br>800 IU/d<br><br>Placebo	ARR	Overall, 12 studies:<br>MD -0.04 (-0.17, 0.09)*	High
						Vitamin D vs. placebo,<br>4 studies:<br>MD 0.00 (-0.10, 0.10)*	
					EDSS	Overall, 5 studies:<br>MD -0.04 (-0.19, 0.03)*	
					Number of new T2 MRI lesions	Overall, 5 studies:<br>MD -0.74 (1.41, -0.06)*	
						Vitamin D vs. placebo,<br>3 studies:	

Meta-analyses of RCTs							
Author, year	**Included studies (n)**	**Participants (n), gender, age**	**Vitamin D dose**	**Control/ Comparator**	**Outcome**	**Results/ Summary statistics (95% CI)**	**AMSTAR 2**
			2857 IU/d (12 mth)	Placebo		MD -0.77 (-1.37, -0.17)*	
			7143 IU (24 mth)<br><br>14,007 IU/d (12 mth)<br><br>7143 IU (12 mth)<br><br>7143 IU (6 mth)<br><br>5000 IU or 10,000 IU/d (6 mth)<br><br><u>Calcitriol</u><br>20 IU/d (12 mth)<br><br><u>Alfacalcidol</u><br>40 IU/d (6 mth)	Placebo<br><br>Placebo<br><br>Placebo<br><br>Placebo<br><br>Placebo<br><br><br>Placebo<br><br><br>Placebo	Number of new Gadolinium-enhancing MRI lesions	Overall, 5 studies:<br>MD -0.14 (-0.56, 0.29)*<br><br>*values of effect estimates (MD and CI) were roughly assessed from depicted forest plots	
Jagannath et al. 2018 [62]	12	n= 933 participants with RRMS (464 treatment group, 469 control group)<br>Range: 23 to 232<br><br>Both sexes<br>Age: 18-60 yr	<u>Vitamin D_2</u><br>6000 IU/d (6 mth)<br><br><u>Vitamin D_3</u><br>50,000 IU/5 d (3 mth)<br><br>40,000 IU/d (28 wk), then 10,000 IU/d (12 wk), then down-titrated to 0 IU/d + 1200 mg calcium/d (52 wk)	1000 IU/d<br><br><br>Placebo<br><br>4000 IU + 1200 mg calcium/d	ARR	Rate difference at 52 weeks follow-up:<br>-0.05 (-0.17, 0.07)<br>(5 studies)	High
					EDSS	MD at 52 weeks follow-up:<br>-0.25 (-0.61, 0.10)<br>(5 studies)	
					Gadolinium-enhancing T1 lesions	MD at 52 weeks follow-up:<br>0.02 (-0.45, 0.48)<br>(2 studies)	

Meta-analyses of RCTs							
Author, year	**Included studies (n)**	**Participants (n), gender, age**	**Vitamin D dose**	**Control/ Comparator**	**Outcome**	**Results/ Summary statistics (95% CI)**	**AMSTAR 2**
			50,000 IU/wk (from 12 to 16 weeks' gestation until delivery; 6 mth)<br><br>800 IU/d (tablet) + 75,000 IU/3 wk (solution) (=4370 IU/d; 1 yr)<br><br>6670 IU/d (4 wk), then 14,007 IU/d (44 wk)<br><br>20,000 IU/wk (96 wk)<br><br>300,000 IU/mth (i.m. injection; 6 mth)<br><br>20,000 IU/wk (12 mth)<br><br>10,000 IU/d + multivitamin + 1000mg calcium/d (6 mth)<br><br><u>Calcitriol</u><br>10 IU/d (2 wk), then 20 IU/d (12 mth)<br><br><u>Alfacalcidol</u><br>40 IU/d (6 mth)	Routine care<br><br>800 IU/d (tablet) + placebo (solution)<br><br>Placebo<br><br>Placebo<br><br>Placebo<br><br>Placebo<br><br>400 IU/d + multivitamin + 1000mg calcium/d<br><br>Placebo<br><br>Placebo	Serious adverse events<br><br>Minor adverse events	Risk difference: 0.01 (-0.03, 0.04) (8 studies)<br><br>Risk difference: 0.02 (-0.02, 0.06) (8 studies)	

Meta-analyses of RCTs							
Author, year	**Included studies (n)**	**Participants (n), gender, age**	**Vitamin D dose**	**Control/ Comparator**	**Outcome**	**Results/ Summary statistics (95% CI)**	**AMSTAR 2**
Zheng et al. 2018 [63]	6	n= 337 participants (169 treatment group/ 168 control group)<br><br>Both sexes (90 men/ 247 women)<br><br>Age: NR	<u>Vitamin D_3</u><br>40,000 IU/d (28 wk), then 10,000 IU/d (12 wk), then 0 IU/d (52wk) +1200 mg calcium/d<br><br>4370 IU/d (12 mth)<br><br>20,000 IU/wk +500 mg calcium/d (96 wk)<br><br>300,000 IU/mth (i,m., 6 mth)<br><br>800,000/wk (12 mth)<br><br><u>Calcitriol</u><br>10 IU/d (2 wk), then 20 IU/d (12 mth)	≤ 4000 IU/d<br><br>800 IU/d<br><br>500 mg calcium/d<br><br>Placebo<br><br>Placebo<br><br>Placebo	EDSS	MD -0.01 (-0.34, 0.33) (6 studies)	Moderate
					ARR	MD 0.05 (0.01, 0.10) (5 studies)	
Hempel et al. 2017 [64]	5	n= 295 participants<br><br>Both sexes<br>Age: ≥ 18 yr (2 studies: NR)	<u>Vitamin D_3</u><br>escalating doses up to 40,000 IU/d (28 wk), then 10,000 IU/d, then 0 IU/d + 1200 mg Calcium/d (52 wk)<br><br>20,000 IU/wk (96 wk)<br><br>300,000 IU/mth (i.m. injection; 6 mth)	Placebo	EDSS	SMD -0.15 (-0.33, 0.02)	Moderate

Meta-analyses of RCTs							
Author, year	**Included studies (n)**	**Participants (n), gender, age**	**Vitamin D dose**	**Control/ Comparator**	**Outcome**	**Results/ Summary statistics (95% CI)**	**AMSTAR 2**
			20,000 IU/wk (1 yr)<br><br>Calcitriol<br>low dose (12 yr)				
James et al. 2013 [65]	5	n= 254 participants (129 high-dose treated MS patients, 125 controls)<br><br>Sex: NR<br>Age: ≥ 15 yr	Vitamin D_3<br>40,000 IU/d (28 wk), then 10,000 IU/d (12 wk), then 0 IU/d (52 wk)<br><br>20,000 IU/week + 500 mg calcium/d (96 wk)<br><br>20,000 IU/wk (1 yr)<br><br>Vitamin D_2<br>13,000 IU/d (6 mth)<br><br>Calcitriol<br>10 IU/d (2 wk), then 20 IU/d (12 mth)	4000 IU/d if desired<br><br>500 mg calcium/d<br><br>Placebo<br><br>1000 IU/d<br><br>Placebo	Relative risk of relapse	Vitamin D vs. control<br>OR 0.98 (0.44, 2.17)	Low

Table S23: Systematic Reviews of RCTs – MS

**Author, year**	**Included studies (n)**	**Participants (n), gender, age**	**Vitamin D dose**	**Control/ Comparator**	**Outcome**	**Results**	**AMSTAR 2**
Berez-owska et al. 2019 [66]	10	n= 627 participants with RRMS (321 treatment group, 264 control group)<br><br>Both sexes (164 men/463 women)<br><br>Age: ≥ 18 yr	<u>Vitamin D3</u><br>20,000 IU/wk (96 wk)<br>75,000 IU/every 3 wk + 800 IU/d (total 4, 370 IU/d; 48 wk)<br>20,000 IU/d (48 wk)<br>50,000 IU/5 d (12 wk)<br>20,000 IU/wk (96 wk)<br>50,000 IU/5 d (12 wk)<br>7000 IU/d (4 wk), then 14,000 IU/d (48 wk)<br>10,000 IU/d (24 wk)<br>20,000/wk (48 wk)<br><u>Calcitriol</u><br>10 IU/d (2 wk), then 20 IU/d (48 wk)	 Placebo<br>Placebo + 800 IU/d<br>Placebo<br>Placebo<br>Placebo<br>Placebo<br>Placebo<br>400 IU/d<br>Placebo<br><br>Placebo	Immunological and inflammatory markers	Improvements in IFN-gamma, IL-17A, IL-9, IL-10, 17+CD4+ T cells, CD161+CD4+ T cells and effector memory CD4+ T cells, the proportion of central memory CD4+ T cells and naive CD4+ T in the intervention group (3/7 studies).<br><br>The majority of studies showed no effect of vitamin D supplementation on immunological and inflammatory markers compared to placebo/low dose vitamin D.	Moderate
					ARR	No significant difference between groups (4/4 studies).	
					EDSS	Benefit in EDSS score after vitamin D supplementation (1/5 studies).	
					Safety and Tolerability	No significant differences between intervention and control/placebo group nor were any of the adverse events serious in either group (4 studies).	

Author, year	Included studies (n)	Participants (n), gender, age	Vitamin D dose	Control/ Comparator	Outcome	Results	AMSTAR 2
Iacopetta et al. 2018 [67]	9 (8 RCTs and 1 non-blinded clinical trial)	n= 560 participants<br><br>Sex and Age: NR	Vitamin D_3<br>50,000/w (i.m. injection)<br><br>50,000 IU/wk (12 mth)<br><br>20,000 IU/wk (12 mth)<br><br>300,000 IU/mth im. injection (6 mth)<br><br>Escalating doses up to 20 IU/d (12 mth)<br><br>4370 IU/d (12 mth)<br><br>20,000 IU/wk (18 mth)<br><br>Vitamin D_2<br>12,000 IU/d (6 mth)<br><br>Alfacalcidol<br>40 IU/d (6 mth)	No injection<br><br>Placebo<br><br>Placebo<br><br>Placebo<br><br>Placebo<br><br>800 IU/d<br><br>Placebo<br><br>1000 IU/d<br><br>Placebo	MS risk	In patients with optic neuritis (associated with MS) who supplemented vitamin D_3 MS risk reduction was 68.4% (1 study).	Low
					EDSS	After 8 weeks of treatment with vitamin D, MS patients had a significant reduction in the mean EDSS scores (1 study).<br>No effect on EDSS score after 6 and 12 months of treatment with vitamin D_3 (3 studies).	
					Relapse rate	No effect on relapse rate with supplementation of vitamin D_3 (3 studies).	
					FIS score	Decreased mean relative FIS score compared to placebo (1 study).	
					MRI disease activity	Supplementation of vitamin D_3 reduced MRI disease activity (1 study).<br>Supplementation of vitamin D_2 had no effect on MRI lesions (1 study).	
Bagur et al. 2017 [68]	7	n= 267 participants<br><br>Both sexes<br>Age: ≥ 18 yr	Vitamin D<br>1000 IU/d (48 wk)<br><br>4000-40,000 IU/d (28 wk)	NR	MRI disease activity	Reduction in brain lesions (4 studies).<br><br>Reduction of MRI disease activity (1 study).	Low

Author, year	Included studies (n)	Participants (n), gender, age	Vitamin D dose	Control/ Comparator	Outcome	Results	AMSTAR 2
			20,000 IU/d (12 wk)<br>200-10,200 IU/d (72 wk)<br>2800 IU/d (96 wk)<br>2800 IU/d (12 wk)<br>7000 IU/d (12 wk)		EDSS<br><br>ARR	Reduced disease activity measured by EDSS (1 study).<br><br>No effect on relapse rate (1 study).	
Autier et al. 2014 [24]	6	n= 241 participants<br><br>Sex and age: NR	Vitamin D range: 2840-32,000 IU/d (6-24 mth)	NR	15 different outcomes assessed by trials (e.g. relapse, disability)	None of the trials showed significant improvements.	Low
Ganesh et al. 2013 [69]	7	n= 363 participants<br><br>Both sexes<br>Age: NR	Vitamin D_2<br>6000 IU (frequency: NR, 6 mth)<br><br>Vitamin D_3<br>40,000 IU/d (52 wk)<br>300,000 IU/mth (i.m. injection; 6 mth)<br>20,000 IU/w (96 wk)<br>800 IU +75,000 IU/3 wk (1yr)<br>20,000 IU (frequency: NR,1yr)	1000 IU (frequency: NR)<br><br>NR<br>NR<br>NR<br>800 IU/3 wk<br>NR	EDSS<br><br>Gadolinium-enhancing lesions change in volume of T2 lesions<br><br>ARR	No effect (3 studies)<br><br>No effect (2 studies); lower increase in T2 burden of disease in vitamin D group (1 study)<br><br>No effect (3 studies)	High

Author, year	Included studies (n)	Participants (n), gender, age	Vitamin D dose	Control/ Comparator	Outcome	Results	AMSTAR 2
			Calcitriol<br>Up to 20 IU/d<br>(duration: NR)	NR			
Pozuelo-Moyano et al. 2013 [70]	5	n= 265 participants (131 treatment group/ 134 control group)<br><br>Sex: NR<br>Age:≥ 15 yr	Vitamin D_2<br>1000 IU + high-dose supplement/d (6 mth)<br><br>Vitamin D_3<br>300,000 IU/mth (6 mth)<br><br>20,000 IU/wk (2 yr)<br><br>20,000 IU/wk (1 yr)<br><br>Calcitriol<br>10 IU/d (2 wk), then 20 IU/d (1 yr)	1000 IU/d + placebo<br><br>NR<br><br>NR	EDSS, MSFC	No significant difference (3 studies).<br>Follow-up EDSS after adjustment for baseline EDSS was higher for high-dose vitamin D_2 than for low-dose vitamin D_2 (1 study).<br>Significant reduction in EDSS (1 study), but due to the small sample size the trial was not powered to address clinical outcomes.	High
					ARR	No significant difference between treatment and control group (3 studies).<br>4 relapses with high-dose vitamin D_2 vs. none with low-dose vitamin D_2 (1 study)	
					T2 lesion load and new T2 or T1 Gadolinium-enhancing lesions	Significant reduction in the number of T1 enhancing lesions and trends in MRI burden of disease (1 study).	

Table S24: Systematic Reviews of prospective cohort studies – MS

**Systematic Reviews of prospective cohort studies**							
**Author, year**	**Included studies (n)**	**Participants (n), gender, age**	**Vitamin D dose**	**Control/ Comparator**	**Outcome**	**Results**	**AMSTAR 2**
Iacopetta et al. 2018 [67]	5	n= 717 participants<br><br>Sex and Age: NR	25(OH)D	-	MS risk	Higher levels of 25(OH)D were associated with lower incidence of MS and MS-related disability in women. Every 10 nmol/l increase of 25(OH)D reduced the MS risk by 19% (1 study).<br><br>Women supplemented with vitamin D had a 40% lower risk of developing MS vs. women with not supplement (1 study).<br><br>Increasing 25(OH)D was associated with lower relapse rate; each 10 nmol/l increase in 25(OH)D the risk was reduced by 9% after adjusting for age and sex (1 study).<br><br>Higher reported sun exposure, rather than 25(OH)D levels were associated with less depressive symptoms and fatigue in MS patients (1 study).	Low
Autier et al. 2014 [24]	3	n= 917 participants cases= 257	25(OH)D		Risk of relapse. disability	Decreases in risk of relapse and disability with high 25(OH)D concentrations in MS patients (2 studies).	Low

Systematic Reviews of prospective cohort studies							
**Author, year**	**Included studies (n)**	**Participants (n), gender, age**	**Vitamin D dose**	**Control/ Comparator**	**Outcome**	**Results**	**AMSTAR 2**
		Sex and age: NR				No association reported (1 study).	
Ganesh et al. 2013 [69]	5	n= 903 participants<br><br>Both sexes<br>Age: NR	25(OH)D		Risk of relapse	Inverse association between 25(OH)D levels and relapse risk (2 studies).<br><br>25(OH)D was associated with lower relapse risk only in those on IFN-β (1 study).<br><br>Lower 25(OH)D levels during pregnancy or post-partum were not associated with increased risk of post-partum relapse (in birth cohort).<br>No association of 25(OH)D levels and relapse risk (2 studies).	High
					Exacerbation rate	Exacerbation rate decreased with each doubling of 25(OH)D levels.	
					Clinical or radiological variables	Each 10 ng/mL increase in 25(OH)D was associated with lower risk of new T2 lesion (1 study).<br><br>No association of 25(OHD) levels with MRI lesions.	

Systematic Reviews of prospective cohort studies							
**Author, year**	**Included studies (n)**	**Participants (n), gender, age**	**Vitamin D dose**	**Control/ Comparator**	**Outcome**	**Results**	**AMSTAR 2**
					EDSS	EDSS progression was not associated with 25(OH)D levels (1 study).<br><br>Each 10 ng/mL increase in 25(OH)D levels was associated with lower subsequent disability.	

Table S25: Meta-analysis of prospective cohort studies – T1DM

Author, year	Included studies (n)	Participants (n), gender, age	Vitamin D dose + insulin	Control/ Comparator	Outcome	Results/ Summary statistics (95% CI)	AMSTAR 2
Dong et al. 2013 [71]	2 cohort studies<br><br>6 case-control studies	Cohort studies: n=10,657<br><br>Case-control studies: n=8103 (1860 cases and 6243 controls)<br><br>Both sexes<br>Age: 0-31	Cohort studies: Questionnaire or FFQ + 25(OH)D<br><br>Case-control studies: Questionnaire or interview	NR	Risk of developing T1DM	Inverse association between vitamin D intake and risk of T1DM (5/8 studies).<br><br>OR = 0.71 (0.51, 0.98)<br>(2 case-control + 6 cohort studies)<br><br>Subgroup analysis by study design:<br><br>OR = 0.68 (0.49–0.94)<br>(6 case-control studies)<br><br>RR = 0.62 (0.11–3.45)<br>(2 cohort studies)	Low

Table S26: Systematic Reviews of RCTs – T1DM

**Systematic Reviews of RCTs**							
**Author, year**	**Included studies (n)**	**Participants (n), gender, age**	**Vitamin D dose**	**Control/ Comparator**	**Outcome**	**Results**	**AMSTAR 2**
Gregoriou et al. 2017 [72]	7	n= 287 participants (newly diagnosed with T1D within a period of 4 wk to 1 yr)<br><br>Both sexes<br>Age: 5-38 yr	<u>Vitamin D_3</u><br>2000 IU/d (18 mth)<br><br>70 IU/kg body weight/d (12 mth)<br><br><u>Calcitriol</u><br>10 IU/on alternate days (1 yr)<br><br>10IU/d (2 yr)<br><br>10 IU/d (9 mth)<br><br><u>Alfacalcidol</u><br>20 IU/d (1 yr)<br><br>10 IU/once or twice daily (based on serum Ca; 6 mth)	Placebo<br><br>Placebo<br><br>NR<br><br>NR<br><br>Placebo<br><br>NR<br><br>Placebo	Changes in daily insulin doses (IU/d)	Insulin doses were significantly lower (treatment vs. control) after 3 and 6 months, but no effect was seen at 12 months (1 study, calcitriol)<br><br>Daily insulin doses were comparable between groups after 9 and 24 months (2 studies, calcitriol)<br><br>Daily insulin doses were significantly different in the between-subject comparison, with lower values in (1 study, alfacalcidol)<br><br>Daily insulin doses were significantly increased in CG, while no change was observed in treatment group (1 study, vitamin D_3)	High
					changes in glycaemic indices (HbA1c, FCP, SCP)	No effect on HbA1c levels during or after treatment (1 study, calcitriol)<br><br>The cumulative incidence of progression to undetectable levels of FCP during 18 months	

Systematic Reviews of RCTs							
**Author, year**	**Included studies (n)**	**Participants (n), gender, age**	**Vitamin D dose**	**Control/ Comparator**	**Outcome**	**Results**	**AMSTAR 2**
						of monitoring was lower (treatment vs. control; 1 study, vitamin D_3)<br><br>Within-subject comparisons showed that the differences in FCP between TG and CG were highest at 3 and 6 months of treatment. FCP levels were reduced in treatment vs. control (1 study, alfacalcidol)<br><br>FCP levels decreased significantly in CG between baseline and months 6 and 12 of therapy, but no changes were observed in TG. Also, FCP levels were maintained or increased (treatment vs. control; 1 study, alfacalcidol)<br><br>SCP increase in the first 12 months and reduced decline after 18 months (treatment vs. control; 1 study, vitamin D_3)	
Antico et al. 2012 [73]	2	n= 51 cases (diagnosed with T1DM) Sexes and age: NR	<u>Alfacalcidol</u><br>20 IU/d (duration: NR)	NR	Insulin requirement<br>β-cell function	Reduction of insulin requirement and protection of β-cell function	Low

References

1. Jolliffe, D.A.; Greenberg, L.; Hooper, R.L.; Griffiths, C.J.; Camargo, C.A.; Kerley, C.P.; Jensen, M.E.; Mauger, D.; Stelmach, I.; Urashima, M.; et al. Vitamin D supplementation to prevent asthma exacerbations: a systematic review and meta-analysis of individual participant data. Lancet Respir Med. 2017, 5, 881–890.
2. Vahdaninia, M.; Mackenzie, H.; Helps, S.; Dean, T. Prenatal intake of vitamins and allergic outcomes in the offspring: a systematic review and meta-analysis. J Allergy Clin Immunol Pract 2017, 5, 771-778.e5.
3. Martineau, A.R.; Cates, C.J.; Urashima, M.; Jensen, M.; Griffiths, A.P.; Nurmatov, U.; Sheikh, A.; Griffiths, C.J. Vitamin D for the management of asthma. Cochrane Database Syst Rev 2016, 9, CD011511.
4. Luo, J.; Liu, D.; Liu, C.-T. Can vitamin D supplementation in addition to asthma controllers improve clinical outcomes in patients with asthma? A meta-analysis. Medicine (Baltimore) 2015, 94, e2185.
5. Riverin, B.D.; Maguire, J.L.; Li, P. Vitamin D supplementation for childhood asthma: a systematic review and meta-analysis. PLoS One 2015, 10, e0136841.
6. Xiao, L.; Xing, C.; Yang, Z.; Xu, S.; Wang, M.; Du, H.; Liu, K.; Huang, Z. Vitamin D supplementation for the prevention of childhood acute respiratory infections: a systematic review of randomised controlled trials. Br J Nutr 2015, 114, 1026–1034.
7. Fares, M.M.; Alkhaled, L.H.; Mroueh, S.M.; Akl, E.A. Vitamin D supplementation in children with asthma: a systematic review and meta-analysis. BMC Res Notes 2015, 8.
8. Pojsupap, S.; Iliriani, K.; Sampaio, T.Z.A.L.; O'Hearn, K.; Kovesi, T.; Menon, K.; McNally, J.D. Efficacy of high-dose vitamin D in pediatric asthma: a systematic review and meta-analysis. J Asthma 2015, 52, 382–390.
9. Shen, S.-Y.; Xiao, W.-Q.; Lu, J.-H.; Yuan, M.-Y.; He, J.-R.; Xia, H.-M.; Qiu, X.; Cheng, K.K.; Lam, K.B.H. Early life vitamin D status and asthma and wheeze: a systematic review and meta-analysis. BMC Pulm Med 2018, 18, 120.
10. Pacheco-González, R.M.; García-Marcos, L.; Morales, E. Prenatal vitamin D status and respiratory and allergic outcomes in childhood: a meta-analysis of observational studies. Pediatr Allergy Immunol 2018, 29, 243–253.
11. Song, H.; Yang, L.; Jia, C. Maternal vitamin D status during pregnancy and risk of childhood asthma: a meta-analysis of prospective studies. Mol Nutr Food Res 2017, 61.
12. Feng, H.; Xun, P.; Pike, K.; Wills, A.K.; Chawes, B.L.; Bisgaard, H.; Cai, W.; Wan, Y.; He, K. In utero exposure to 25-hydroxyvitamin D and risk of childhood asthma, wheeze, and respiratory tract infections: a meta-analysis of birth cohort studies. J Allergy Clin Immunol 2017, 139, 1508–1517.
13. Wei, Z.; Zhang, J.; Yu, X. Maternal vitamin D status and childhood asthma, wheeze, and eczema: a systematic review and meta-analysis. Pediatr Allergy Immunol 2016, 27, 612–619.
14. Man, L.; Zhang, Z.; Zhang, M.; Zhang, Y.; Li, J.; Zheng, N.; Cao, Y.; Chi, M.; Chao, Y.; Huang, Q.; et al. Association between vitamin D deficiency and insufficiency and the risk of childhood asthma: Evidence from a meta-analysis. Int. J. Clin Exp Med 2015, 8, 5699–5706.
15. Cassim, R.; Russell, M.A.; Lodge, C.J.; Lowe, A.J.; Koplin, J.J.; Dharmage, S.C. The role of circulating 25 hydroxyvitamin D in asthma: A systematic review. Allergy 2015, 70, 339–354.
16. Jat, K.R.; Khairwa, A. Vitamin D and asthma in children: a systematic review and meta-analysis of observational studies. Lung India 2017, 34, 355–363.

17. Harvey, N.C.; Holroyd, C.; Ntani, G.; Javaid, K.; Cooper, P.; Moon, R.; Cole, Z.; Tinati, T.; Godfrey, K.; Dennison, E.; et al. Vitamin D supplementation in pregnancy: a systematic review. Health Technol Assess 2014, 18, 1–190.
18. Rajabbik, M.H.; Lotfi, T.; Alkhaled, L.; Fares, M.; El-Hajj Fuleihan, G.; Mroueh, S.; Akl, E.A. Association between low vitamin D levels and the diagnosis of asthma in children: a systematic review of cohort studies. Allergy Asthma Clin Immunol 2014, 10.
19. Jolliffe, D.A.; Greenberg, L.; Hooper, R.L.; Mathyssen, C.; Rafiq, R.; Jongh, R.T. de; Camargo, C.A.; Griffiths, C.J.; Janssens, W.; Martineau, A.R. Vitamin D to prevent exacerbations of COPD: systematic review and meta-analysis of individual participant data from randomised controlled trials. Thorax 2019.
20. Zhu, M.; Wang, T.; Wang, C.; Ji, Y. The association between vitamin D and COPD risk, severity, and exacerbation: an updated systematic review and meta-analysis. Int J Chron Obstruct Pulmon Dis 2016, 11, 2597–2607.
21. Zhu, B.; Zhu, B.; Xiao, C.; Zheng, Z. Vitamin D deficiency is associated with the severity of COPD: a systematic review and meta-analysis. Int J Chron Obstruct Pulmon Dis 2015, 10, 1907–1916.
22. Autier, P.; Mullie, P.; Macacu, A.; Dragomir, M.; Boniol, M.; Coppens, K.; Pizot, C.; Boniol, M. Effect of vitamin D supplementation on non-skeletal disorders: a systematic review of meta-analyses and randomised trials. Lancet Diabetes Endocrinol 2017.
23. Autier, P.; Boniol, M.; Pizot, C.; Mullie, P. Vitamin D status and ill health: a systematic review. Lancet Diabetes Endocrinol 2014, 2, 76–89.
24. Ferrari, R.; Caram, L.M.O.; Tanni, S.E.; Godoy, I.; Rupp de Paiva, S.A. The relationship between Vitamin D status and exacerbation in COPD patients- a literature review. Respir Med 2018, 139, 34–38.
25. Martineau, A.R.; Jolliffe, D.A.; Greenberg, L.; Aloia, J.F.; Bergman, P.; Dubnov-Raz, G.; Esposito, S.; Ganmaa, D.; Ginde, A.A.; Goodall, E.C.; et al. Vitamin D supplementation to prevent acute respiratory infections: individual participant data meta-analysis. Health Technol Assess 2019, 23, 1–44.
26. Das, R.R.; Singh, M.; Naik, S.S. Vitamin D as an adjunct to antibiotics for the treatment of acute childhood pneumonia. Cochrane Database Syst Rev 2018, 7, CD011597.
27. Vuichard Gysin, D.; Dao, D.; Gysin, C.M.; Lytvyn, L.; Loeb, M. Effect of vitamin D_3 supplementation on respiratory tract infections in healthy individuals: a systematic review and meta-analysis of randomized controlled trials. PLoS One 2016, 11, e0162996.
28. Yakoob, M.Y.; Salam, R.A.; Khan, F.R.; Bhutta, Z.A. Vitamin D supplementation for preventing infections in children under five years of age. Cochrane Database Syst Rev 2016, 11, CD008824.
29. Bergman, P.; Lindh, A.U.; Björkhem-Bergman, L.; Lindh, J.D. Vitamin D and respiratory tract infections: a systematic review and meta-analysis of randomized controlled trials. PLoS One 2013, 8, e65835.
30. Mao, S.; Huang, S. Vitamin D supplementation and risk of respiratory tract infections: a meta-analysis of randomized controlled trials. Scand J Infect Dis 2013, 45, 696–702.
31. Charan, J.; Goyal, J.P.; Saxena, D.; Yadav, P. Vitamin D for prevention of respiratory tract infections: a systematic review and meta-analysis. J Pharmacol Pharmacother 2012, 3, 300–303.
32. Das, R.R.; Singh, M.; Panigrahi, I.; Naik, S.S. Vitamin d supplementation for the treatment of acute childhood pneumonia: a systematic review. ISRN Pediatr 2013, 2013, 459160.
33. Jolliffe, D.A.; Griffiths, C.J.; Martineau, A.R. Vitamin D in the prevention of acute respiratory infection: systematic review of clinical studies. J Steroid Biochem Mol Biol 2013, 136, 321–329.

34. Fried, D.A.; Rhyu, J.; Odato, K.; Blunt, H.; Karagas, M.R.; Gilbert-Diamond, D. Maternal and cord blood vitamin D status and childhood infection and allergic disease: a systematic review. Nut Rev 2016, 74, 387–410.
35. Goodwill, A.M.; Szoeke, C. A systematic review and meta-analysis of the effect of low vitamin D on cognition. J Am Geriatr Soc 2017, 65, 2161–2168.
36. Annweiler, C.; Llewellyn, D.J.; Beauchet, O. Low serum vitamin D concentrations in Alzheimer's disease: a systematic review and meta-analysis. J Alzheimers Dis 2013, 33, 659–674.
37. Chen, H.; Xue, W.; Li, J.; Fu, K.; Shi, H.; Zhang, B.; Teng, W.; Tian, L. 25-hydroxyvitamin D levels and the risk of dementia and Alzheimer's disease: a dose-response meta-analysis. Front Aging Neurosci 2018, 10, 368.
38. Jayedi, A.; Rashidy-Pour, A.; Shab-Bidar, S. Vitamin D status and risk of dementia and Alzheimer's disease: a meta-analysis of dose-response. Nutr Neurosci 2018, 15, 1–10.
39. Sommer, I.; Griebler, U.; Kien, C.; Auer, S.; Klerings, I.; Hammer, R.; Holzer, P.; Gartlehner, G. Vitamin D deficiency as a risk factor for dementia: a systematic review and meta-analysis. BMC Geriatr 2017, 17.
40. Cao, L.; Tan, L.; Wang, H.-F.; Jiang, T.; Zhu, X.-C.; Lu, H.; Tan, M.-S.; Yu, J.-T. Dietary patterns and risk of dementia: a systematic review and meta-analysis of cohort studies. Mol Neurobiol 2016, 53, 6144–6154.
41. Shen, L.; Ji, H.-F. Vitamin D deficiency is associated with increased risk of Alzheimer's disease and dementia: evidence from meta-analysis. Nutr J 2015, 14.
42. Etgen, T.; Sander, D.; Bickel, H.; Sander, K.; Förstl, H. Vitamin D deficiency, cognitive impairment and dementia: a systematic review and meta-analysis. Dement. Geriatr Cogn Disord 2012, 33, 297–305.
43. Lerner, P.P.; Sharony, L.; Miodownik, C. Association between mental disorders, cognitive disturbances and vitamin D serum level: current state. Clin Nutr ESPEN 2018, 23, 89–102, doi:10.1016/j.clnesp.2017.11.011.
44. Balion, C.; Griffith, L.E.; Strifler, L.; Henderson, M.; Patterson, C.; Heckman, G.; Llewellyn, D.J.; Raina, P. Vitamin D, cognition, and dementia: a systematic review and meta-analysis. Neurology 2012, 79, 1397–1405.
45. Killin, L.O.J.; Starr, J.M.; Shiue, I.J.; Russ, T.C. Environmental risk factors for dementia: a systematic review. BMC Geriatr. 2016, 16.
46. van der Schaft, J.; Koek, H.L.; Dijkstra, E.; Verhaar, H.J.J.; van der Schouw, Y.T.; Emmelot-Vonk, M.H. The association between vitamin D and cognition: a systematic review. Ageing Res Rev 2013, 12, 1013–1023.
47. Vellekkatt, F.; Menon, V. Efficacy of vitamin D supplementation in major depression: a meta-analysis of randomized controlled trials. J Postgrad Med 2018.
48. Gowda, U.; Mutowo, M.P.; Smith, B.J.; Wluka, A.E.; Renzaho, A.M.N. Vitamin D supplementation to reduce depression in adults: meta-analysis of randomized controlled trials. Nutrition 2015, 31, 421–429.
49. Spedding, S. Vitamin D and depression: a systematic review and meta-analysis comparing studies with and without biological flaws. Nutrients 2014, 6, 1501–1518.
50. Shaffer, J.A.; Edmondson, D.; Wasson, L.T.; Falzon, L.; Homma, K.; Ezeokoli, N.; Li, P.; Davidson, K.W. Vitamin D supplementation for depressive symptoms: a systematic review and meta-analysis of randomized controlled trials. Psychosom Med 2014, 76, 190–196.
51. Li, G.; Mbuagbaw, L.; Samaan, Z.; Falavigna, M.; Zhang, S.; Adachi, J.D.; Cheng, J.; Papaioannou, A.; Thabane, L. Efficacy of vitamin D supplementation in depression in adults: a systematic review. J Clin Endocrinol Metab 2014, 99, 757–767.

52. Wang, J.; Liu, N.; Sun, W.; Chen, D.; Zhao, J.; Zhang, W. Association between vitamin D deficiency and antepartum and postpartum depression: a systematic review and meta-analysis of longitudinal studies. Arch Gynecol Obstet 2018, 298, 1045–1059.
53. Ju, S.-Y.; Lee, Y.-J.; Jeong, S.-N. Serum 25-hydroxyvitamin D levels and the risk of depression: a systematic review and meta-analysis. J Nutr Health Aging 2013, 17, 447–455.
54. Anglin, R.E.S.; Samaan, Z.; Walter, S.D.; McDonald, S.D. Vitamin D deficiency and depression in adults: systematic review and meta-analysis. Br J Psychiatry 2013, 202, 100–107.
55. Aghajafari, F.; Letourneau, N.; Mahinpey, N.; Cosic, N.; Giesbrecht, G. Vitamin D deficiency and antenatal and postpartum depression: a systematic review. Nutrients 2018, 10.
56. Föcker, M.; Antel, J.; Ring, S.; Hahn, D.; Kanal, Ö.; Öztürk, D.; Hebebrand, J.; Libuda, L. Vitamin D and mental health in children and adolescents. Eur Child Adolesc Psychiatry 2017, 26, 1043–1066.
57. Sarris, J.; Murphy, J.; Mischoulon, D.; Papakostas, G.I.; Fava, M.; Berk, M.; Ng, C.H. Adjunctive nutraceuticals for depression: a systematic review and meta-analyses. Am J Psychiatry 2016, 173, 575–587.
58. Trujillo, J.; Vieira, M.C.; Lepsch, J.; Rebelo, F.; Poston, L.; Pasupathy, D.; Kac, G. A systematic review of the associations between maternal nutritional biomarkers and depression and/or anxiety during pregnancy and postpartum. J Affect Disord 2018, 232, 185–203.
59. Amini, S.; Jafarirad, S.; Amani, R. Postpartum depression and vitamin D: a systematic review. Crit Rev Food Sci Nutr 2018.
60. Sparling, T.M.; Nesbitt, R.C.; Henschke, N.; Gabrysch, S. Nutrients and perinatal depression: a systematic review. J Nutr Sci 2017, 6, e61.
61. McLaughlin, L.; Clarke, L.; Khalilidehkordi, E.; Butzkueven, H.; Taylor, B.; Broadley, S.A. Vitamin D for the treatment of multiple sclerosis: a meta-analysis. J Neurol 2018, 265, 2893–2905.
62. Jagannath, V.A.; Filippini, G.; Di Pietrantonj, C.; Asokan, G.V.; Robak, E.W.; Whamond, L.; Robinson, S.A. Vitamin D for the management of multiple sclerosis. Cochrane Database Syst Rev 2018, 9, CD008422.
63. Zheng, C.; He, L.; Liu, L.; Zhu, J.; Jin, T. The efficacy of vitamin D in multiple sclerosis: a meta-analysis. Mult Scler Relat Disord 2018, 23, 56–61.
64. Hempel, S.; Graham, G.D.; Fu, N.; Estrada, E.; Chen, A.Y.; Miake-Lye, I.; Miles, J.N.V.; Shanman, R.; Shekelle, P.G.; Beroes, J.M.; et al. A systematic review of modifiable risk factors in the progression of multiple sclerosis. Mult Scler 2017, 23, 525–533.
65. James, E.; Dobson, R.; Kuhle, J.; Baker, D.; Giovannoni, G.; Ramagopalan, S.V. The effect of vitamin D-related interventions on multiple sclerosis relapses: a meta-analysis. Mult Scler 2013, 19, 1571–1579.
66. Berezowska, M.; Coe, S.; Dawes, H. Effectiveness of vitamin D supplementation in the management of multiple sclerosis: a systematic review. Int J Mol Sci 2019, 20.
67. Iacopetta, K.; Collins-Praino, L.E.; Buisman-Pijlman, F.T.A.; Liu, J.; Hutchinson, A.D.; Hutchinson, M.R. Are the protective benefits of vitamin D in neurodegenerative disease dependent on route of administration? A systematic review. Nutr Neurosci 2018.
68. Bagur, M.J.; Murcia, M.A.; Jiménez-Monreal, A.M.; Tur, J.A.; Bibiloni, M.M.; Alonso, G.L.; Martínez-Tomé, M. Influence of diet in multiple sclerosis: a systematic review. Adv Nutr 2017, 8.
69. Ganesh, A.; Apel, S.; Metz, L.; Patten, S. The case for vitamin D supplementation in multiple sclerosis. Mult Scler Relat Disord 2013, 2, 281–306.
70. Pozuelo-Moyano, B.; Benito-León, J.; Mitchell, A.J.; Hernández-Gallego, J. A systematic review of randomized, double-blind, placebo-controlled trials examining the clinical efficacy of vitamin D in multiple sclerosis. Neuroepidemiology 2013, 40, 147–153.

71. Dong, J.-Y.; Zhang, W.-G.; Chen, J.J.; Zhang, Z.-L.; Han, S.-F.; Qin, L.-Q. Vitamin D intake and risk of type 1 diabetes: a meta-analysis of observational studies. Nutrients 2013, 5, 3551–3562.
72. Gregoriou, E.; Mamais, I.; Tzanetakou, I.; Lavranos, G.; Chrysostomou, S. The effects of vitamin D supplementation in newly diagnosed type 1 diabetes patients: systematic review of randomized controlled trials. Rev Diabet Stud 2017, 14, 260–268.
73. Antico, A.; Tampoia, M.; Tozzoli, R.; Bizzaro, N. Can supplementation with vitamin D reduce the risk or modify the course of autoimmune diseases? A systematic review of the literature. Autoimmun Rev 2012, 12, 127–136.
